# Formin-like protein 2 promotes cell proliferation by a p27-related mechanism in human breast cancer cells

**DOI:** 10.1186/s12885-021-08533-w

**Published:** 2021-06-30

**Authors:** Xinyan Jiao, Bo Wang, Chen Feng, Shaoran Song, Bixia Tian, Can Zhou, Xiaoqian Gao, Wei Sun, Peijun Liu

**Affiliations:** 1 Center for Translational Medicine, the First Affiliated Hospital of Xi’an Jiaotong University, 277 West Yanta Road, 710061 Xi’an, Shaanxi People’s Republic of China; 2 Key Laboratory for Tumor Precision Medicine of Shaanxi Province, the First Affiliated Hospital of Xi’an Jiaotong University, 710061 Xi’an, Shaanxi People’s Republic of China; 3 Department of Oncology, Shaanxi Provincial Corps Hospital, 710061 Xi’an, Shaanxi People’s Republic of China; 4 Department of Breast Surgery, the First Affiliated Hospital of Xi’an Jiaotong University, 710061 Xi’an, Shaanxi People’s Republic of China

**Keywords:** FMNL2, Cell proliferation, Breast cancer, p27, Nuclear

## Abstract

**Background:**

Breast cancer is the leading cause of cancer-related deaths in females worldwide. Formin-like protein 2 (FMNL2) is a member of formin family that governs cytokinesis, cell polarity, morphogenesis and cell division. To our knowledge, the function of FMNL2 in breast cancer proliferation still remains uncovered.

**Methods:**

Tumor immune estimation resource (TIMER) analysis was used to detect the correlation between FMNL2 and Ki67 in breast cancer tissues. Quantitative real-time transcription polymerase chain reaction (qRT-PCR) and western blotting were performed to analyze the expression in human breast cancer cells. Moreover, RNA interference (RNAi) and plasmids were performed to silence and overexpress FMNL2 and p27. The CCK8, MTT, cell counting, colony formation, and 5-ethynyl-2-deoxyuridine (EdU) incorporation assays were used to detect cell proliferation, respectively. Flow cytometry analysis was used to detect cell cycle distribution. Further, the distribution of p27 was examined using immunofluorescence.

**Results:**

We found that FMNL2 expression was positively associated with Ki67 among collected breast cancer tissues and in TCGA database. Compared to lower proliferative cells MCF7 and T47D, FMNL2 was overexpressed in highly proliferative breast cancer cells MDA-MB-231, BT549 and SUM159, accompanied by reduced levels of p27 and p21, and elevated CyclinD1 and Ki67 expression. FMNL2 silencing significantly inhibited the cell proliferation of MDA-MB-231 and BT549 cells. Meanwhile, FMNL2 overexpression distinctly promoted the cell proliferation of MCF7 cells. Furthermore, FMNL2 suppressed the nuclear levels of p27 and promoted p27 proteasomal degradation in human breast cancer cells. The ubiquitination of p27 was inhibited by FMNL2 silencing in BT549 cells. Besides, p27 silencing markedly elevated Ki67 expression and cell viability, which could be blocked by additionally FMNL2 silencing in MDA-MB-231 and BT549 cells. Furthermore, overexpression of p27WT significantly reversed the increased levels of FMNL2 and Ki67, cell viability and cell cycle progression induced by FMNL2 overexpression in MCF7 cells. More importantly, compared to p27WT group, those effects could be significantly reversed by p27△NLS overexpression.

**Conclusions:**

These results demonstrated that FMNL2 promoted cell proliferation partially by reducing p27 nuclear localization and p27 protein stability in human breast cancer cells, suggesting the pivotal role of FMNL2 in breast cancer progression.

**Supplementary Information:**

The online version contains supplementary material available at 10.1186/s12885-021-08533-w.

## Background

Breast cancer is one of the most common occurring malignancies in women worldwide with an increasing annual incidence [1]. Studies have revealed that breast cancer is a heterogeneous disease that possesses various histopathological features, genetic markers, and diverse prognostic outcomes [2]. Early detection and diagnosis have markedly ameliorated the treatment options and survival of breast cancer patients. Notably, multiple genes have been regarded to be implicated in cell proliferation and breast cancer progression, and represent to be promising effective therapeutic targets [3, 4]. Thus, it is urgent to explore novel cancer proliferation biomarkers for formulating more individualized treatments, and predicting cancer prognosis.

Formins are key regulators of the actin cytoskeletal remodeling, and thus have been commonly associated with tumor progression [5]. In mammals, 15 formins are identified and grouped into eight different subfamilies [6, 7]. The formin-like (FMNL) subfamily is metazoan specific and contains three genes, formin-like protein 1 (FMNL1), formin-like protein 2 (FMNL2), and formin-like protein 3 (FMNL3) in vertebrates [8]. Particularly, FMNL2 is disorderly expressed in multiple types of cancers, for instance, gastric cancer [9], colorectal cancer [10], melanoma [11], oral squamous cell carcinoma [12], and hepatocellular carcinoma [13]. To our knowledge, the role of FMNL2 in breast cancer progression still remains uncovered.

In addition, FMNL2 is a catalyst of linear actin polymerization and therefore has been involved in a variety of actin-dependent cellular processes including adhesion, cytokinesis, filopodium formation, division and especially cellular polarity [9, 14–17]. Breast cancer is closely associated with cell polarity, and disrupting polarity proteins affects many stages of breast cancer progression from initiation through metastasis [18]. We speculated that FMNL2 might be implicated in the malignant progression of human breast cancer. A good deal of research has indicated that the high mitotic rate and the increased proliferation rate have been described as the most well-known characteristics of malignant tumors [19]. In this study, here we mainly focused on the effect of FMNL2 on cell proliferation and related mechanisms in human breast cancer cells.

## Methods

### Tissue specimens

A total of 114 breast cancer samples were obtained from the First Affiliated Hospital of Xi’an Jiaotong University (Xi’an, China). None of patients received chemotherapy before operation. All patients provided their written informed consent to participate in this study. All specimens were independently evaluated by 2 pathologists. This study was performed in accordance with the Declaration of Helsinki and approved by the Ethics Committee of the First Affiliated Hospital of Xi’an Jiaotong University (no. XJTU1AF2018LSK-127).

### Cell lines and plasmids

All the human breast cancer cell lines were obtained from Shanghai Institute of Biochemistry and Cell Biology (Chinese Academy of Sciences, Shanghai, China). All cell lines were authenticated by short tandem repeat (STR) DNA fingerprinting and tested for mycoplasma contamination. The MDA-MB-231 and MCF7 cells were maintained in Dulbecco’s Modified Eagle’s Medium (DMEM; (Hyclone, Logan, UT, USA), BT549 and T47D cells were maintained in Roswell Park Memorial Institute (RPMI)-1640 (Hyclone, Logan, UT, USA), MCF10A and SUM159 cells were cultured in DMEM/F12 (1:1) medium (Hyclone), with all recommended supplements, respectively. All cultures were maintained at 37 °C in a humidified incubator in an atmosphere of 5% CO_2_.

The sequence of nuclear localization signal of p27 was retrieved from UniProt database (https://www.uniprot.org/uniprot/). Then we obtained full-length p27 wild-type cDNA fragment using the primers, namely p27WT-F: 5′-CGCGGATCCATGTCAAACGTGCGAGTGTCT-3′ and p27WT-R: 5′-CGGAATTCTTACGTTTGACGTCTTCTGAGGCC-3′. To generate the full-length p27△NLS that localized exclusively in cell cytosol, we introduced the primers, namely p27△NLS-F: 5′-GAGCAATGCGCAGGAATAAGGACAGAAGAAAATGTTTCAGACGGT-3′ and p27△NLS-R: 5′-ACCGTCTGAAACATTTTCTTCTGTCCTTATTCCTGCGCATTGCTC-3′. First, using full-length p27 wild-type cDNA fragment as template, two different overlapping fragments of p27 genes were obtained by PCR amplifications with primers p27WT-F and p27△NLS-R, p27△NLS-F and p27WT-R, respectively. Second, 1 μL of each of the two fragments were pooled together and a second round PCR was conducted with primers p27WT-F and p27WT-R to generate the full-length p27△NLS cDNA fragment. Subsequently, the p27 wild-type and p27△NLS fragments were excised by BamHI/EcoRI restriction enzyme digestion and cloned into the pCMV-N-Flag vector to generate pCMV-N-Flag-p27 wild-type (referred herein as p27WT) and pCMV-N-Flag-p27△NLS (referred herein as p27△NLS) plasmids.

### Small interfering RNA (siRNA) and cell transfection

MDA-MB-231 and BT549 cells were transfected with corresponding siRNAs using Lipofectamine 2000 (Invitrogen, Carlsbad, CA, USA) following the manufacturer’s protocol. The siRNA sequences for human FMNL2 and p27, and the negative control siRNA (NC siRNA) were synthesized (GenePharma Biotechnology, Shanghai, China). Target sequences of oligonucleotides were as follows: siFMNL2–1, 5′-GCGUGUUCAAGAAUCUACATT-3′ and 5′-UGUAGAUUCUUGAACACGCTT-3′; siFMNL2–2, 5′-GCCCUUGUCUUAGAACUGUTT-3′ and 5′-ACAGUUCUAAGACAAGGGCTT-3′; sip27, 5′-GCAACCGACGAUUCUUCUATT-3′ and 5′-UAGAAGAAUCGUCGGUUGCTT-3′; NC siRNA, 5′-UUCUCCGAACGUGUCACGUTT-3′ and 5′-ACGUGACACGUUCGGAGAATT-3′. Forty-eight hours later, cultured cells were performed for indicated assays.

### Quantitative real-time reverse transcription polymerase chain reaction (qRT-PCR) assay

Briefly, total RNA was extracted using the RNAfast 200 kit (Shanghai Fastagen Biotech Ltd. Co., China), and cDNA was synthesized with PrimeScript RT Master Mix (Takara Biotechnology, Dalian, China) following the manufacturer’s protocol. The qRT-PCR was conducted with TB Green Premix Ex Taq II (Takara Biotechnology, Dalian, China). The primers were synthesized (Sangon Biotech, Shanghai, China) and listed as follows: FMNL2-F, 5′-TAATCAGCATTAGCATTTCTGAGG-3′, and FMNL2-R, 5′-AGGAGAGTAAGGCCAGGTTCC-3′; CDK4-F, 5′-ACGGGTGTAAGTGCCATCTG-3′, and CDK4-R, 5′-TGGTGTCGGTGCCTATGGGA-3′; CDK6-F, 5′-CCACTGAGGTTAGAGCCATC-3′, and CDK6-R, 5′-CGAATGCGTGGCGGAGATC-3′; GAPDH-F, 5′-AGAAGGCTGGGGCTCATTTG-3′, and GAPDH-R, 5′-AGGGGCCATCCACAGTCTTC-3′. Each experiment was carried out in triplicate and standardized to GAPDH levels.

### 3-(4,5-dimethylthiazol-2-yl)-2,5-diphenyltetrazolium bromide dye (MTT) assay

Cultured cells (1 × 10^3^ cells/well) were seeded into 96-well plate (Corning Inc., Corning, NY, USA) in triplicate after cell transfection. After an incubation period of 1, 2, 3, 4 and 5 days, cells were stained with 20 μL of MTT (5 mg/mL; Sigma, St. Louis, MO, USA) for 4 h at 37 °C. Then culture medium was removed and 150 μL dimethyl sulfoxide (Sigma, St. Louis, MO, USA) was added. Afterwards, the cells were agitated on orbital shaker for 15 min, and the absorbance was determined at 490 nm using a Benchmark microplate reader (Bio-Rad, Hercules, CA, USA).

### Cell counting Kit-8 (CCK8) assay

Cultured cells (1 × 10^3^ cells/well) were seeded into 96-well plate (Corning Inc., Corning, NY, USA) in triplicate after cell transfection. After an incubation period of 1, 2, 3, 4 and 5 days, 10 μL of CCK8 solution (Dojindo Molecular Technologies Inc., Kumamoto, Japan) was added and incubated for 2 h at 37 °C. Then the absorbance was detected at 450 nm using a Benchmark microplate reader (Bio-Rad, Hercules, CA, USA).

### Cell counting assay

Cultured cells (5 × 10^3^ cells/well) were seeded into 24-well plate (Corning Inc., Corning, NY, USA) in triplicate after cell transfection. After trypsinization, an aliquot of the cell suspension was counted in a hemocytometer and the results were expressed as cell number. Proliferation curves were based on cell counting for 5 consecutive days.

### Colony formation assay

After transfection, cultured cells were trypsinized to generate single-cell suspensions and seeded into 6-well plate (Corning Inc., Corning, NY, USA) at 500 cells/well to monitor colony-forming ability. After 2 weeks in culture at 37 °C, the cells were fixed with methanol, stained with crystal violet and then analyzed by Image J software (NIH, Bethesda, MD, USA).

### 5-ethynyl-2-deoxyuridine (EdU) incorporation assay

After transfection, cells were seeded in 96-well plate in triplicate. Then the medium was replaced with fresh medium containing EdU. After culturing for 2 h, EdU detection was also performed according to the protocol using the kFluor555 Click-iT EdU Cell Proliferation Kit (Nanjing KeyGen Biotech. Co. Ltd., Nanjing, China) according to the manufacturer’s instructions. Images were taken by a Leica DMi8 Microscope (Leica, Germany), and the EdU-positive cells were counted. All experiments were performed at least in triplicate.

### Flow cytometry analysis

Cultured cells (2 × 10^5^ cells/well) were seeded in 6-well plate (Corning Inc., Corning, NY, USA). Forty-eight hours after transfection, cells were gathered, fixed in 70% ethanol overnight at 4 °C, and then incubated with 50 μg/mL propidium iodide (PI; Sigma, St. Louis, MO, USA) and 10 μg/mL RNase A (Sigma, St. Louis, MO, USA) for 30 min in the dark. Afterwards, quantitative analysis of DNA content was performed by flow cytometry (BD Biosciences, San Jose, CA, USA), and the results were analyzed using ModFit LT (BD Biosciences).

### CDK4/CyclinD1 kinase activity assay

After transfection for 48 h, cells were processed for CDK4/CyclinD1 kinase activity according to the manufacturer’s instructions (GENMED SCIENTIFICS INC., U.S.A). The absorbance was determined at 340 nm using a Benchmark microplate reader (Bio-Rad, Hercules, CA, USA).

### Western blotting

Cell fractionation assays were performed using Nuclear and Cytoplasmic Extraction Reagents (Pioneer Biotechnology, Xi’an, China) following the manufacturer’s instructions. The co-immunoprecipitation (Co-IP) assay were conducted by a Dynabeads protein G immunoprecipitation kit (Invitrogen, Carlsbad, CA, USA) according to the manufacturer’s instructions. Equal amounts of protein were separated on sodium dodecyl sulfate-polyacrylamide gel electrophoresis (SDS-PAGE) and transferred to polyvinylidene difluoride (PVDF) membranes. Subsequently, the membranes were blocked in 5% nonfat milk and then incubated overnight at 4 °C with the primary antibodies against FMNL2, MCM7, PCNA and p27 (Santa Cruz Biotechnology, Santa Cruz, CA, USA); Ki67 and ERα (Abcam, Cambridge, MA, USA); Ubiquitin and PR (Cell Signaling Technology, USA.); HER2, p21, CyclinD1, Lamin A/C, GAPDH and β-actin (Proteintech, Wuhan, China). After incubation with proper HRP-conjugated secondary antibody, the protein bands were visualized by an enhanced chemiluminescence system (Millipore, Bedford, MA, USA).

### Immunofluorescence

Briefly, cells were fixed, permeabilized, blocked with 5% bovine serum albumin (BSA), and then incubated with p27 antibody. Incubation with secondary antibody Dylight 594 goat anti rabbit IgG (H&L) (EarthOx, E032420–01; San Francisco, CA, USA) was followed by 4′,6-diamidino-2-phenylindole (DAPI) staining. Images were taken by a Leica DMi8 Microscope (Leica, Germany).

### Data processing

Clinical data from 1093 tissue samples in breast cancer patients from The Cancer Genomic Atlas (TCGA) was retrieved from Tumor Immune Estimation Resource (TIMER) (https://cistrome.shinyapps.io/timer/) to study the clinical association between FMNL2 and Ki67 in breast cancer.

### Statistical analysis

Statistical analysis was performed by using a GraphPad Prism version 5.0 software (GraphPad Software, Inc., La Jolla, CA, USA) and SPSS version 20.0 (SPSS, Chicago, IL, USA). All Data were expressed as mean ± standard deviation (SD). Each experiment was repeated at least three times. Correlations between FMNL2 and clinicopathological features of breast cancer patients were analyzed by using Pearson’s chi-squared test. Comparison between two independent groups was made by the Student’s *t*-test and a two-way ANOVA. * *P* < 0.05 was considered as statistically significant.

## Results

### The expression of FMNL2 was associated with cell proliferation in breast cancer

**Fig. 1:**
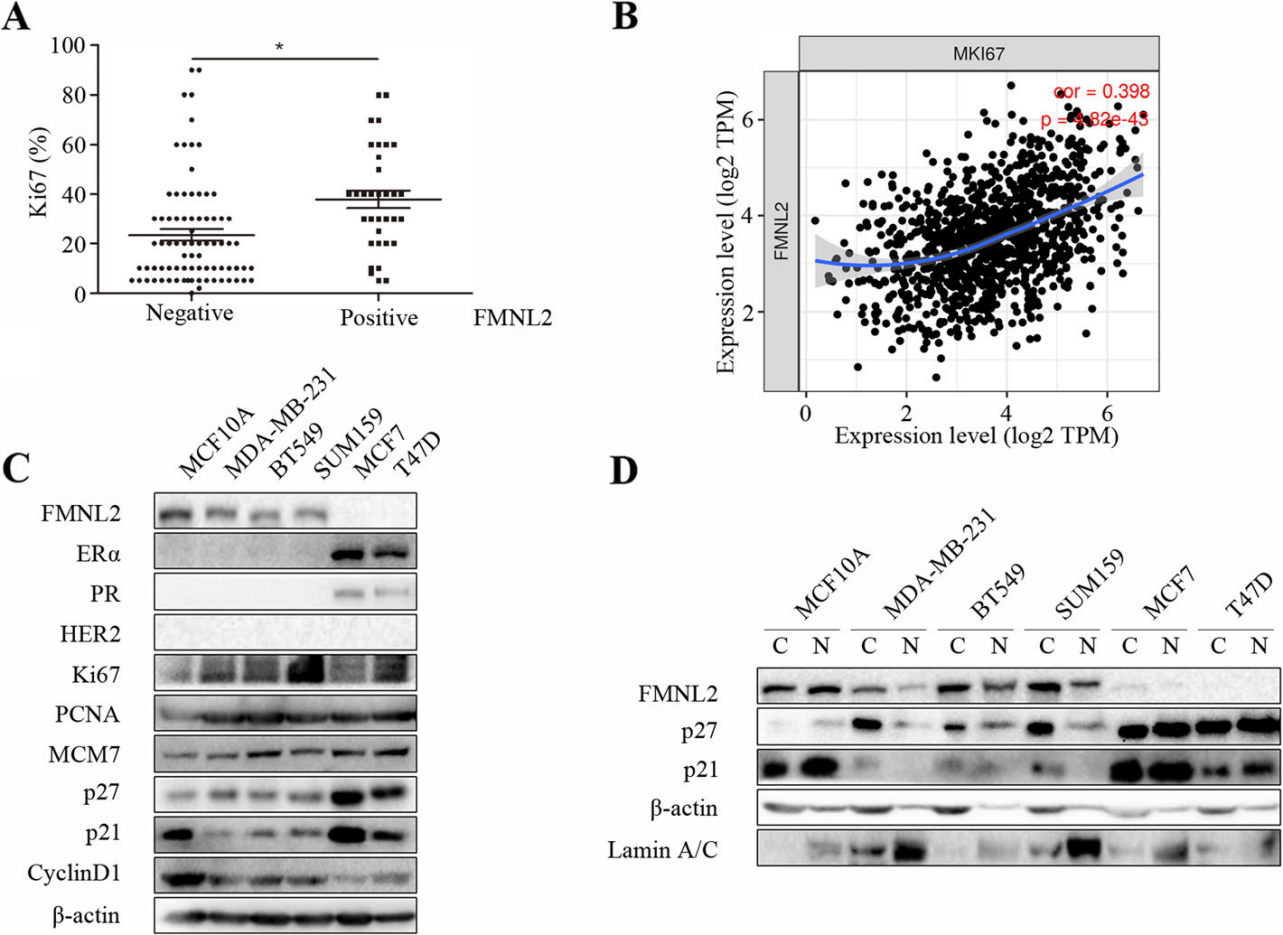
The correlation of FMNL2 with cell proliferation in breast cancer. **a** Association of FMNL2 and Ki67 in tumor tissues from breast cancer patients. **b** The correlation of FMNL2 and Ki67 expression was determined in breast cancer samples using TIMER. **c** and **d** The levels of ERα, PR, HER2, Ki67, PCNA, MCM7, FMNL2, p27, p21, CyclinD1, Lamin A/C, and β-actin proteins were determined by western blotting and representative blots were shown. *: *P* < 0.05 vs FMNL2 negative group

Firstly, we analyzed the clinical relevance between FMNL2 expression and clinicopathological variables in tumor specimens from breast cancer patients. Notably, there was a highly positive association between FMNL2 and Ki67 (Fig. 1a; Table 1; *P* < 0.05). Moreover, there is also a significant positive correlation between FMNL2 and Ki67 in breast cancer samples from the TCGA database (Fig. 1b). Additionally, FMNL2 was negatively correlated with ER status in breast cancer tissues (Table 1; *P* < 0.05). MCF7 and T47D cells were ER-positive, PR-positive and HER2-negative. MDA-MB-231, BT549 and SUM159 cells were ER-negative, PR-negative and HER2-negative. Besides, there are no differences in the expression levels of PCNA and MCM7 in these human breast cancer cell lines, and that FMNL2 expression was markedly related to p27, p21 and CyclinD1 in human breast cancer cells. Compared to lower proliferative cells MCF7 and T47D, highly proliferative breast cancer cells MDA-MB-231, BT549 and SUM159 expressed high levels of FMNL2 along with high proliferative features including enhanced Ki67 and CyclinD1 levels, and reduced levels of p27 and p21 (Fig. 1c). Interestingly, FMNL2 and p27 was mainly expressed in the cytoplasm of MDA-MB-231, BT549 and SUM159 cells; whereas FMNL2 and p27 was mainly located in the nucleus of normal human breast cells MCF10A (Fig. 1d). These results demonstrated that the expression of FMNL2 was associated with cell proliferation in breast cancer, and that the role of FMNL2 and p27 in cell proliferation of human breast cancer might be correlated with its intracellular distribution in the cytoplasm and nucleus.

**Table 1:** The relationship of FMNL2 and clinicopathological variables in breast cancer tissues

Variables	Total No. of patients (*n* = 114)	FMNL2 expression		χ^2^	*P*-value
		Negative (*n* = 80)	Positive (*n* = 34)		
Age				1.565	0.211
< 50	65	47	18		
≥ 50	49	30	19		
histopathological type				0.814	0.665
Invasive ductal carcinoma	111	78	33		
Ductal carcinoma in situ	2	1	1		
Invasive lobular carcinoma	1	1	0		
Clinical stage				5.48	0.065
1	14	12	2		
2	48	37	11		
3	52	31	21		
TNM phase				0.329	0.567
I-II	65	47	18		
III-IV	49	33	16		
Ki67				11.668	0.001*
< 14%	44	39	5		
≥ 14%	70	41	29		
Tumor size				0.495	0.482
< 2 cm	32	24	8		
≥ 2 cm	82	56	26		
ER status				6.327	0.012*
ER (−)	29	15	14		
ER (+)	85	65	20		
PR status				2.173	0.14
PR (−)	42	26	16		
PR (+)	72	54	18		
HER2 status				0.31	0.578
HER2 (−)	36	24	12		
HER2 (+)	78	56	22		

*P < 0.05 indicates a significant association between the variables

### FMNL2 silencing suppressed cell proliferation in human breast cancer cells

**Fig. 2:**
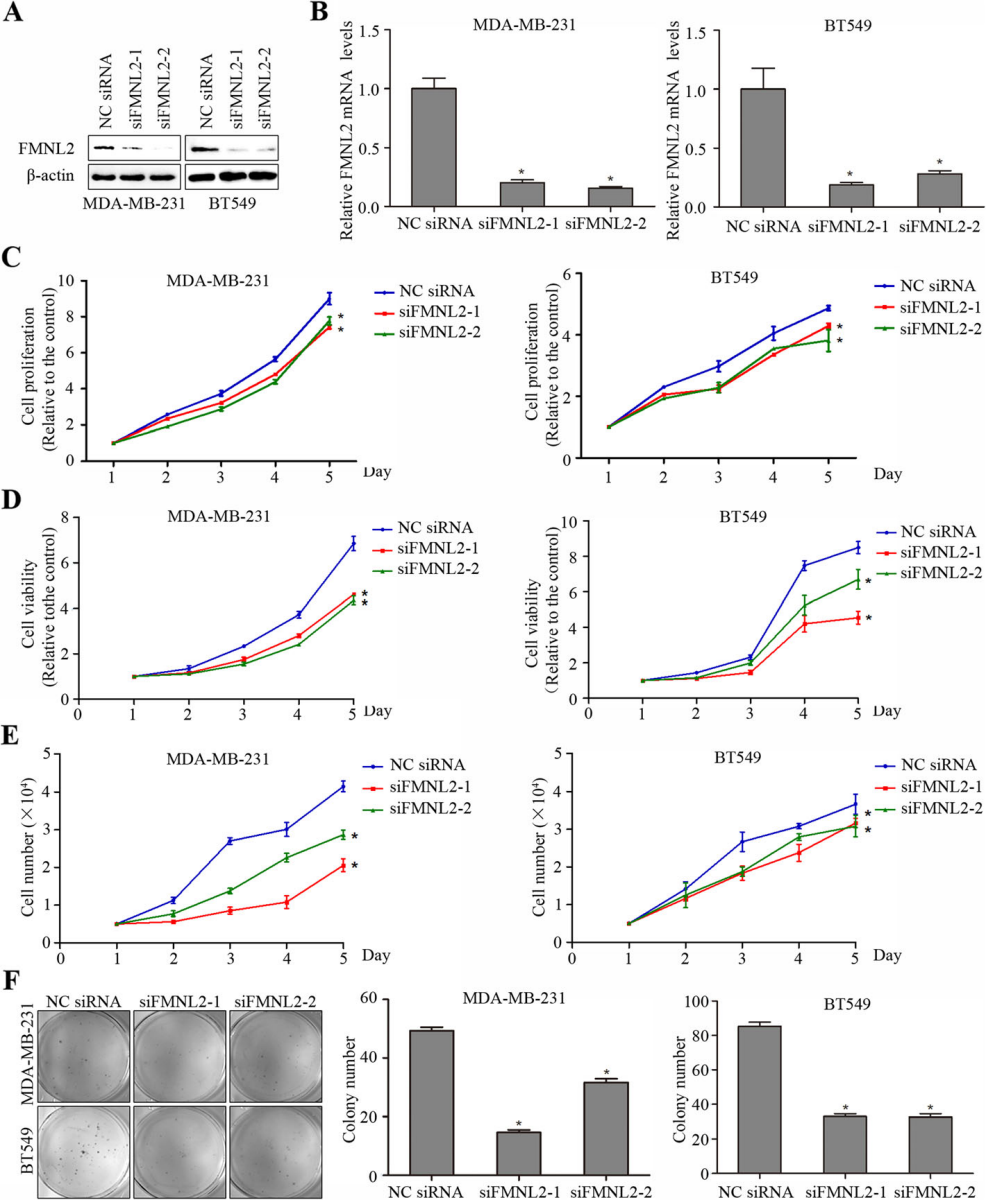
Effects of FMNL2 silencing on cell proliferation in MDA-MB-231 and BT549 cells. After transfection for 48 h, cultured cells were processed for indicated assays. **a** The expression of FMNL2 protein was determined by western blotting analyses. **b** The expression of FMNL2 mRNA was determined by qRT-PCR analyses. **c** The cell proliferation was detected by CCK8 assay. **d** The cell viability was examined by MTT assay. **e** The cell number was determined by cell counting assay. **f** The number of forming colonies was counted and analyzed statistically. *: *P* < 0.05 vs NC siRNA group

Next, we explored whether FMNL2 played a functional role in cell proliferation of breast cancer in vitro. Due to the high expression of FMNL2 in both MDA-MB-231 and BT549 cells, FMNL2 was silenced in these two cell lines for subsequent experiments. After transfection with siRNA specific for FMNL2, the expression of FMNL2 protein and mRNA was significantly decreased in MDA-MB-231 and BT549 cells (Fig. 2a and b). The CCK8 assay and MTT assay showed that both cell proliferation ability and cell viability was significantly suppressed by FMNL2 silencing in MDA-MB-231 and BT549 cells (Fig. 2c and d). The cell counting assay further indicated that FMNL2 silencing markedly lowered the cell number of MDA-MB-231 and BT549 cells (Fig. 2e). Meanwhile, colony formation assay indicated that the number of forming colonies was distinctively reduced by FMNL2 silencing in both MDA-MB-231 and BT549 cells (Fig. 2f). Thus, we may conclude that FMNL2 silencing inhibited cell proliferation in human breast cancer cells.

### FMNL2 silencing inhibited EdU incorporation and induced cell cycle arrest in human breast cancer cells

**Fig. 3:**
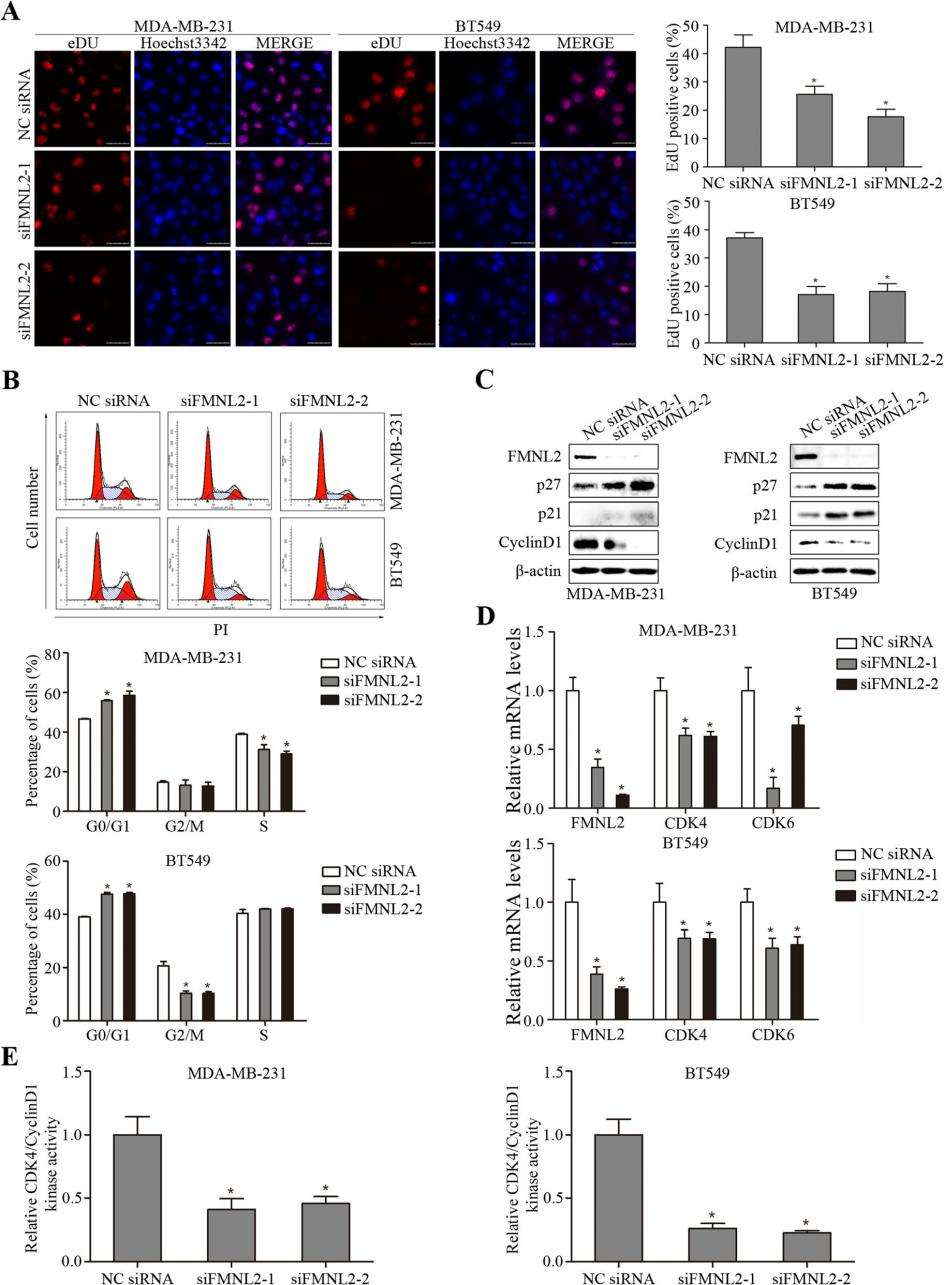
Effects of FMNL2 silencing on cell cycle and proliferation markers in MDA-MB-231 and BT549 cells. After transfection for 48 h, cultured cells were processed for indicated assays. **a** Representative EdU staining images of cells after transfection were shown. **b** Representative percentage of cells in the G0/G1, G2/M and S phases was detected by flow cytometric analysis. **c** The levels of FMNL2, p27, p21, CyclinD1, and β-actin proteins were determined by western blotting and representative blots were shown. **d** The levels of CDK4 and CDK6 mRNA were determined by qRT-PCR. **e** The CDK4/CyclinD1 kinase activity was determined. Scale bar, 50 μm. *: *P* < 0.05 vs NC siRNA group

Then, we evaluated the function of FMNL2 silencing on EdU incorporation and cell cycle distribution in MDA-MB-231 and BT549 cells. Decreased cell proliferation after FMNL2 silencing was confirmed by reduced EdU positive cell rate (Fig. 3a). Expectedly, flow cytometry analysis revealed that FMNL2 silencing caused arrest of cell cycle at G1 phase in MDA-MB-231 and BT549 cells (Fig. 3b). We further assessed whether FMNL2 silencing induced expression changes of the cell cycle regulatory genes and proteins. The results confirmed that FMNL2 silencing enhanced the levels of p21 and p27, but inhibited CyclinD1 levels in MDA-MB-231 and BT549 cells (Fig. 3c). It was also observed that FMNL2 silencing significantly reduced the levels of CDK4 and CDK6 mRNA, and the levels of CDK4/CyclinD1 kinase activity in these two cell lines (Fig. 3d and e). Therefore, our findings demonstrated that FMNL2 silencing inhibited EdU incorporation and induced cell cycle arrest in human breast cancer cells.

### FMNL2 overexpression elevated cell proliferation in human breast cancer cells

**Fig. 4:**
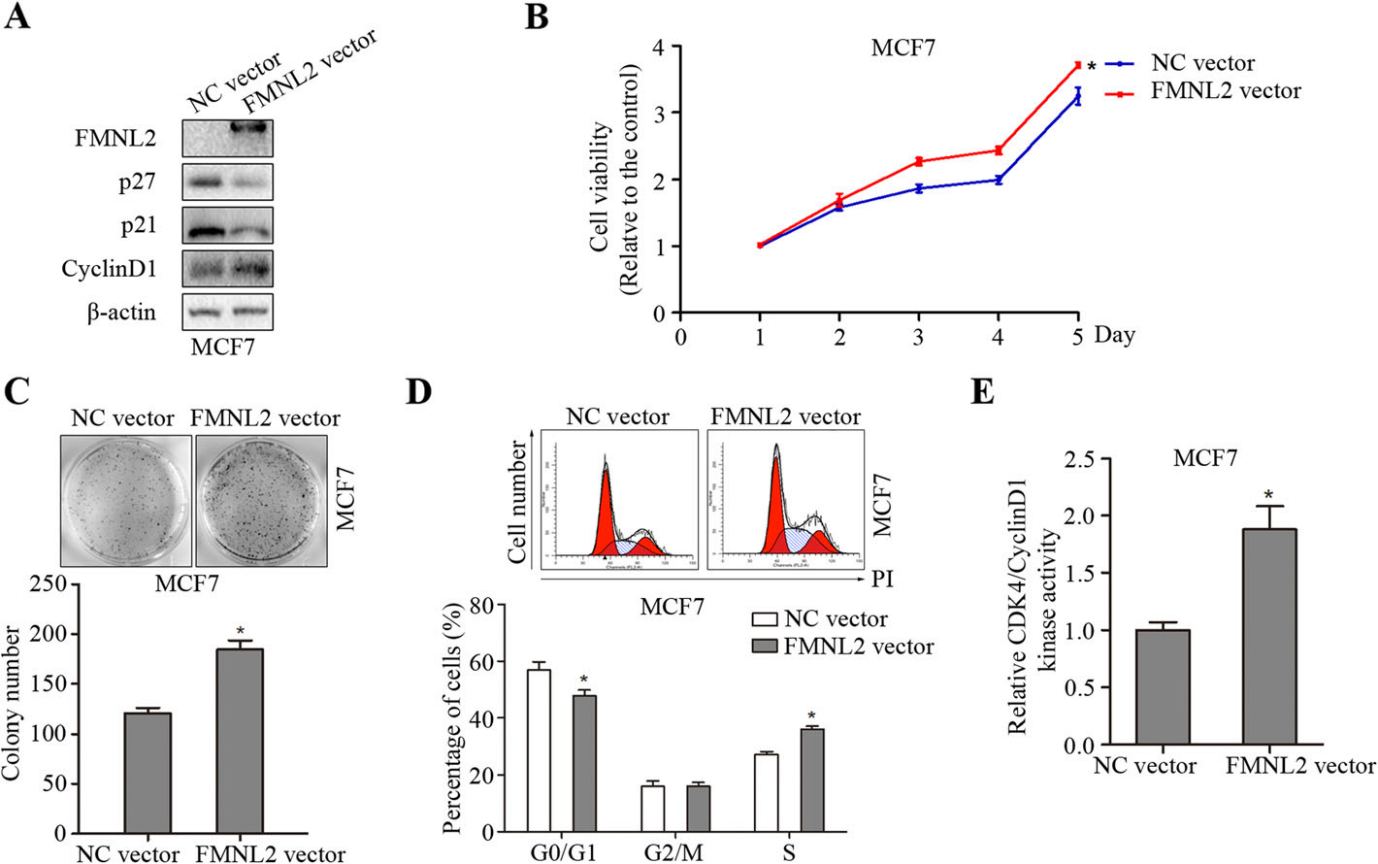
Effects of FMNL2 overexpression on cell proliferation in MCF7 cells. After transfection for 48 h, cultured cells were processed for indicated assays. **a** The levels of FMNL2, p27, p21, CyclinD1, and β-actin proteins were determined by western blotting and representative blots were shown. **b** The cell viability was examined by MTT assay. **c** The number of forming colonies was counted and analyzed statistically. **d** Representative percentage of cells in the G0/G1, G2/M and S phases was detected by flow cytometric analysis. **e** The CDK4/CyclinD1 kinase activity was determined. *: *P* < 0.05 vs NC vector group

Further, we investigated the effect of FMNL2 overexpression on cell proliferation of human breast cancer. FMNL2 overexpression significantly reduced the levels of p21 and p27, but elevated CyclinD1 levels in MCF7 cells (Fig. 4a). The cell viability and the number of forming colonies were remarkably increased by FMNL2 overexpression in MCF7 cells (Fig. 4b and c). Moreover, FMNL2 overexpression promoted cell cycle progression and enhanced CDK4/CyclinD1 kinase activity in MCF7 cells (Fig. 4d and e). Therefore, our results demonstrated that FMNL2 overexpression elevated cell proliferation in human breast cancer MCF7 cells.

### FMNL2 reduced the nuclear p27 levels and promoted p27 degradation in human breast cancer cells

**Fig. 5:**
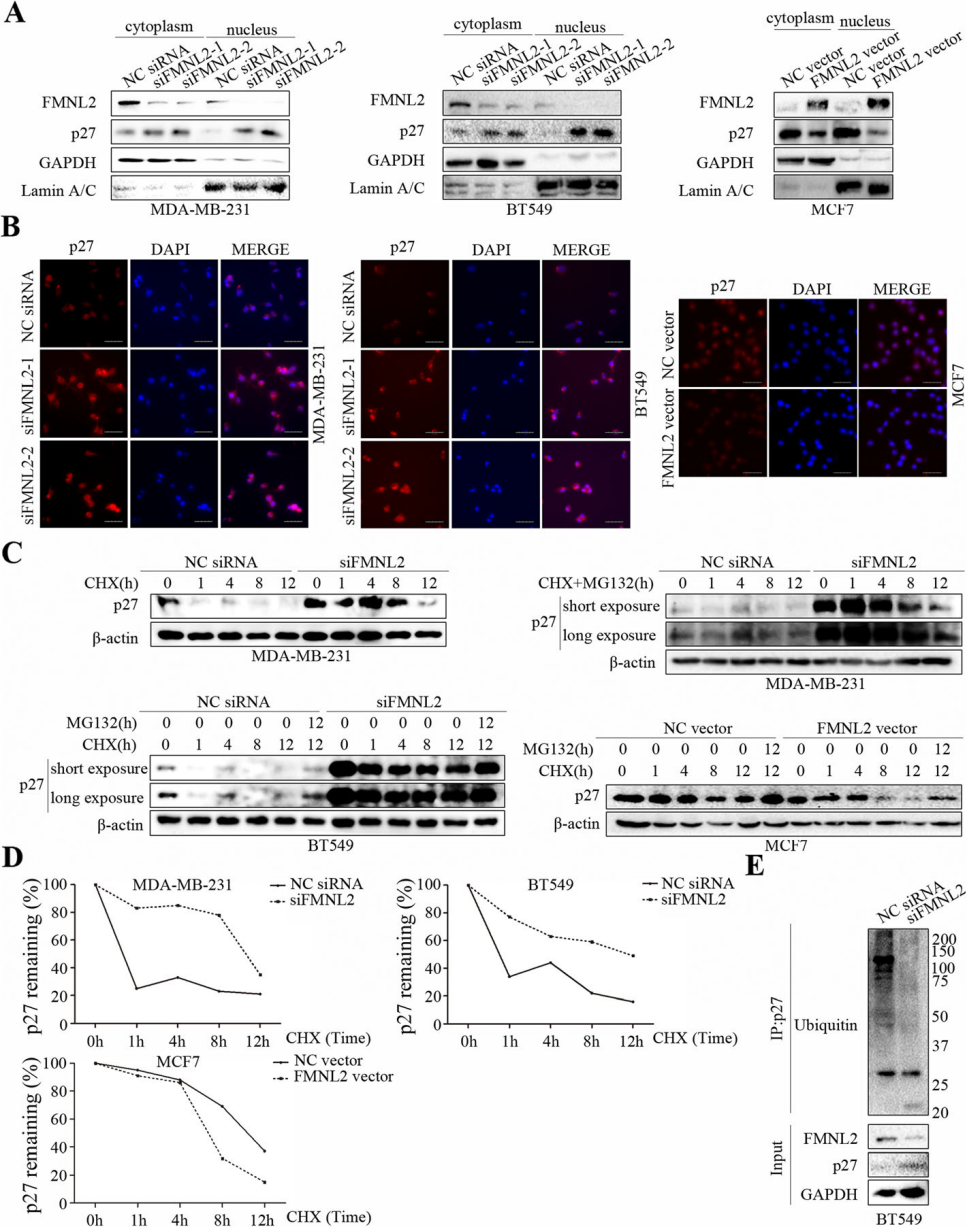
Effects of FMNL2 on the nuclear levels of p27 and the degradation of p27. **a** Cellular fractionation experiments were performed to examine the levels of FMNL2 and p27 protein in the nucleus and cytoplasm. GAPDH and Lamin A/C were used as loading controls. **b** Representative fluorescent images of p27 were displayed. **c** After transfection for 48 h, cultured cells were incubated with cyclohexmide (CHX, 100 μg/mL) or MG132 (5 μM) for the indicated times. The levels of p27 protein were detected by western blotting. **d** A plot of normalized p27 expression is shown. **e** The ubiquitination of p27 was detected by western blotting. Scale bar, 50 μm

To explore the related mechanism implicated in the regulation of cell proliferation by FMNL2, nuclear and cytoplasmic proteins were fractionated to further determine the expression changes. We found that the levels of nuclear and cytoplasmic p27 and especially the nuclear/cytoplasmic protein ratios of p27 were remarkably increased by FMNL2 silencing in MDA-MB-231 and BT549 cells, but decreased by FMNL2 overexpression in MCF7 cells (Fig. 5a). The fluorescence levels of p27 were consistent with the above results (Fig. 5b). To further examine how FMNL2 regulated p27 levels, the half-life of p27 was determined in the presence of a protein synthesis inhibitor cycloheximide. The results showed that FMNL2 silencing significantly extended the half-life of p27 in MDA-MB-231 and BT549 cells, and FMNL2 overexpression shortened the half-life of p27 in MCF7 cells (Fig. 5c and d). Under long exposure, we may clearly observe that the reduced p27 levels caused by cycloheximide could be effectively restored by MG132 in MDA-MB-231 and BT549 cells. MG132 treatment could also restore the decreased p27 levels caused by cycloheximide in MCF7 cells. Furthermore, the ubiquitination of p27 was inhibited by FMNL2 silencing in BT549 cells (Fig. 5e). These data revealed that FMNL2 reduced the nuclear levels of p27 and promoted p27 degradation in human breast cancer cells.

### FMNL2 promoted cell proliferation partially by a p27-related mechanism in human breast cancer cells

**Fig. 6:**
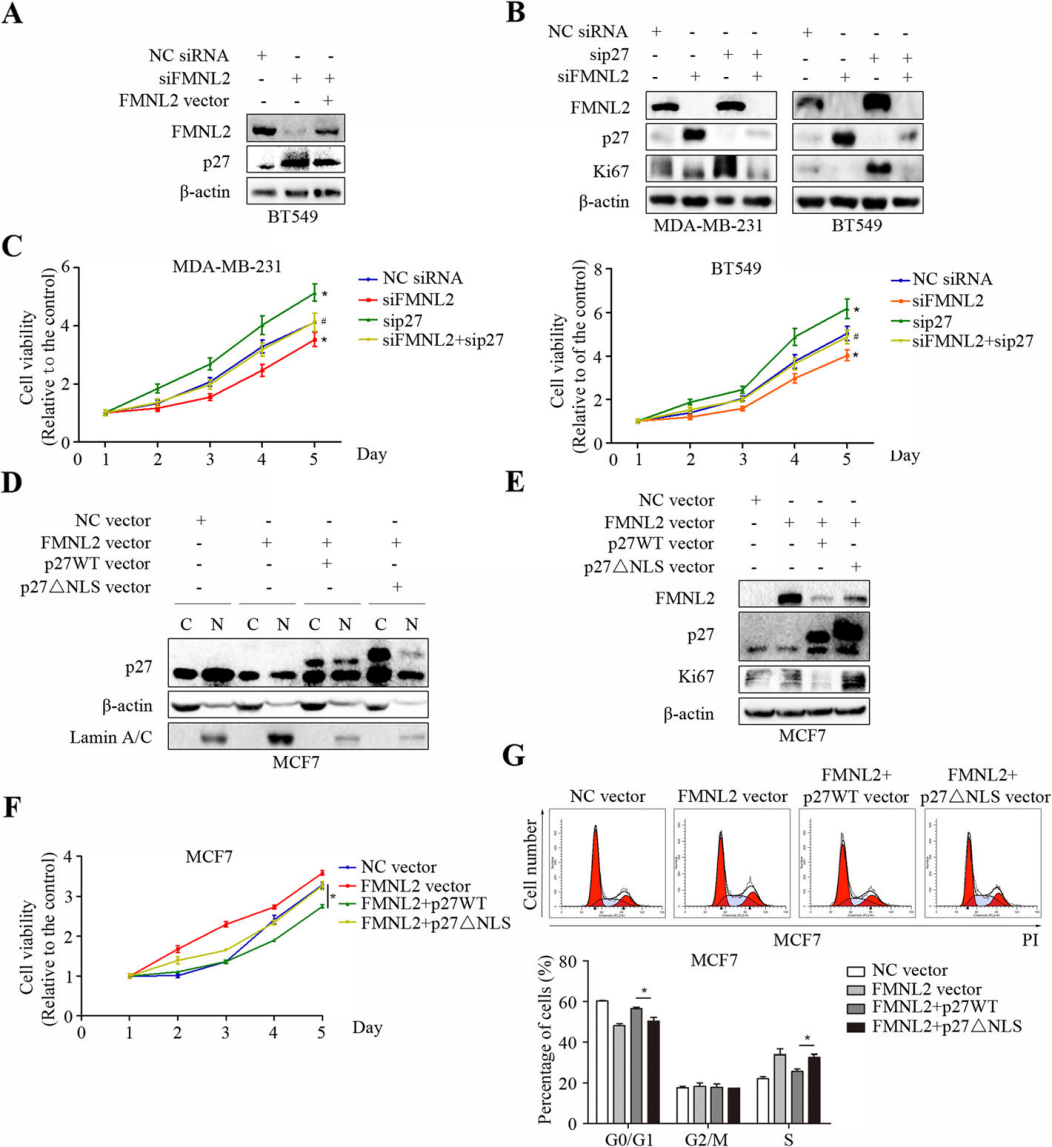
FMNL2 promotes breast cancer cell proliferation partially by a p27-related mechanism. After transfection for 48 h, cultured cells were processed for indicated assays. **a** and **b** The expression of FMNL2, p27 and Ki67 was detected by western blotting. **c** MTT assay was performed at 48 h post-transfection. *: *P* < 0.05 vs NC siRNA group. ^#^: *P* < 0.05 vs sip27 group. **d** and **e** The expression of FMNL2, p27, Lamin A/C and Ki67 was detected by western blotting. **f** MTT assay was performed at 48 h post-transfection. **g** Representative percentage of cells in the G0/G1, G2/M and S phases was detected by flow cytometric analysis. *: *P* < 0.05 vs p27WT vector group

Here, we tested whether the reduced cell proliferation following FMNL2 silencing depended on p27. The results showed that FMNL2 overexpression attenuated the increased levels of p27 caused by FMNL2 silencing in BT549 cells (Fig. 6a). Then we depleted FMNL2 combined with p27 in MDA-MB-231 and BT549 cells. Strikingly, p27 silencing markedly elevated Ki67 expression and cell viability in MDA-MB-231 and BT549 cells, which could be blocked by additionally FMNL2 silencing (Fig. 6b and c). Subsequently, we generated p27WT vector and p27△NLS vector to investigate the effect of nuclear p27 levels on cell proliferation of human breast cancer. Both cytoplasmic and nuclear levels of p27 were overexpressed after transfection of p27WT, whereas the nuclear levels of p27 were just weakly expressed after transfection of p27△NLS in FMNL2-overexpressed MCF7 cells (Fig. 6d). Furthermore, overexpression of p27WT significantly reversed the increased levels of FMNL2 and Ki67, cell viability and cell cycle progression induced by FMNL2 overexpression in MCF7 cells. After FMNL2 overexpression, compared to p27WT group, the reduced levels of FMNL2 and Ki67, the inhibited cell viability and cell cycle progression were significantly reversed by p27△NLS overexpression in MCF7 cells (Fig. 6e, f and g). These results suggested that the role of FMNL2 in breast cancer cell proliferation could at least partially account for p27 intracellular nuclear distribution and p27 protein stability.

## Discussion

Breast cancer is the leading cause of cancer-related deaths in females worldwide [20]. Given that cell proliferation performs a critical role in the progression of breast cancer, it is therefore essential to identify novel potential treatment targets, and explore the regulatory mechanisms of breast cancer cell proliferation, aiming to improve the efficiency of molecular targeted therapies. It is noteworthy that Ki67 is widely used as proliferative index in clinical breast cancer diagnosis and treatment [21–24]. The expression of Ki67 protein is also closely correlated with cell proliferation in breast cancer progression [4]. In this work, we observed a significant positive correlation between FMNL2 and Ki67 in breast cancer samples. Compared to lower proliferative cells MCF7 and T47D, FMNL2 was overexpressed in highly proliferative breast cancer cells MDA-MB-231, BT549 and SUM159, accompanied by reduced levels of p27 and p21, and elevated CyclinD1 and Ki67 expression. These data imply that FMNL2 may function as a positive regulator in breast cancer proliferation. Surprisingly, FMNL2 was also positively expressed in normal human breast MCF10A cells. According to our results, we speculated that the more nuclear localization of p27 and FMNL2 might inhibit the cell proliferation of MCF10A cells. We may infer that the cytoplasm/nucleus distribution of FMNL2 and p27 is associated with the cell proliferation of human breast cancer. Further studies are needed to explore the exact mechanism.

FMNL2 is a member of formin family, which governs cytokinesis, cell polarity, morphogenesis, and cell division [2]. Mounting evidence has shown that FMNL subfamily participates in cell proliferation during cancer progression. FMNL2 knockdown suppresses growth of gastric cancer cells through suppressing internalization of integrins [9]. Additionally, FMNL2 has been regarded as a potential target for abnormal proliferation in colorectal cancer [25, 26]. FMNL1 increases the proliferation of leukemia cells [27], and FMNL3 promotes cell proliferation and clonogenicity in colorectal cancer [28]. However, the function of FMNL2 in breast cancer cell proliferation has not been reported. In this study, FMNL2 was weakly expressed in ER-positive MCF7 and T47D cells, but was positively expressed in ER-negative MDA-MB-231, BT549 and SUM159 cells. Then FMNL2 was silenced in MDA-MB-231 and BT549 cells (ER-negative), and overexpressed in MCF7 cells (ER-positive) for functional experiments. Consistent with previous studies, our experimental results proved that FMNL2 silencing suppressed cell proliferation in MDA-MB-231 and BT549 cells, and FMNL2 overexpression promoted cell proliferation in MCF7 cells. Besides, the expression of FMNL2 was negatively correlated with ER status in breast cancer tissues. Obviously, the results of in vitro assays indicated that the positive role of FMNL2 in cell proliferation of human breast cancer cells was not correlated with ER status. However, the mechanisms underlying how FMNL2 regulates cell proliferation in breast cancer is currently unknown.

Previously, other formins have been reported to alter cell proliferation through their role in cytokinesis [17], microtubule stabilization [29], or through regulation of cell cycle [30]. Specifically, the cyclin-dependent kinases (CDKs) such as CyclinD1, play a vital role in the progression of G1/S cell cycle. It has been well documented that the cyclin-dependent kinase inhibitors (CKIs) such as p27 and p21, can bind and restrain the activity of virtually all cyclin-CDK complexes, resulting in cell cycle arrest at G0/G1 phase [31, 32]. Research has shown that the diaphanous-related formin Dia1 enables Mitf to coordinate actin polymerization with cell cycle progression through mediating p27 stability in melanoma [30]. More importantly, our findings revealed that FMNL2 silencing arrested cell cycle at G1 phase accompanied by enhanced levels of p21 and p27 protein, and decreased levels of CyclinD1 protein, reduction of CDK4 and CDK6 mRNA, as well as decreased CDK4/CyclinD1 kinase activity in both MDA-MB-231 and BT549 cells. Similarly, FMNL2 overexpression promoted cell cycle progression and enhanced CDK4/CyclinD1 kinase activity in MCF7 cells.

According to our results, the critical cell cycle regulator p27 was found to be significantly increased following FMNL2 depletion. It is generally accepted that the inhibitory activity of p27 is largely dependent on their nuclear subcellular localization, which is necessary for their suppressive roles in cell cycle transition [33]. In our study, FMNL2 silencing induced a significant increase in nuclear localization of p27 in MDA-MB-231 and BT549 cells, and FMNL2 overexpression clearly reduced nuclear localization of p27 in MCF7 cells. Interestingly, FMNL2 depletion significantly prolonged the half-life of p27, and FMNL2 overexpression distinctly shortened the half-life of p27. We may infer that FMNL2 decreased the nuclear localization of p27 and the protein stability of p27, thus promoting CDK4/CyclinD1 kinase activity and cell proliferation of human breast cancer cells.

A large body of literature has indicated that p27 degradation occurs mainly through the proteasomal pathway, and proteasomal degradation of the growth suppressor p27 facilitates mammalian cell cycle progression [34, 35]. Furthermore, few studies have addressed the possibility of proteasome-independent mechanisms of p27 proteolysis [36]. Here, our data indicated that the degradation of p27 levels could be effectively restored by MG132, implying that FMNL2 may mainly regulate the proteasomal degradation of p27. Indeed, the ubiquitination of p27 was clearly inhibited by FMNL2 silencing. Additionally, possibility of other degradation pathways may also play a role in p27 degradation including lysosomal degradation. It has been reported that in addition to the proteasome-dependent pathway, SNX6-mediated endolysosomal degradation of p27 also contributes to cell cycle progression in mammalian cells [36]. This requires more research to explore whether lysosomal degradation of p27 was involved in FMNL2-regulated cell proliferation of breast cancer.

Additionally, FMNL2 overexpression could reverse the elevated p27 levels induced by FMNL2 silencing in BT549 cells. We also found that p27 silencing markedly elevated Ki67 expression and cell viability in MDA-MB-231 and BT549 cells, which could be blocked by additionally FMNL2 silencing. Furthermore, overexpression of p27WT significantly reversed the increased levels of FMNL2 and Ki67, cell viability and cell cycle progression induced by FMNL2 overexpression in MCF7 cells. More importantly, compared to p27WT group, those effects could be significantly reversed by p27△NLS overexpression. In all, our data indicated that FMNL2 may promote CDK4/CyclinD1 kinase activity and cell proliferation through inhibition of p27 nuclear localization and p27 protein stability in human breast cancer cells.

## Conclusions

Taken together, these findings strongly reinforced that FMNL2 promoted cell proliferation partially by reducing p27 nuclear localization and p27 protein stability in human breast cancer cells. Our results also suggest that more attention should be paid to the pivotal role of FMNL2 in breast cancer progression, and especially the thorough underlying mechanisms.

## Supplementary Information

**Additional file 1.**

## Data Availability

The datasets used and/or analyzed during the current study are available from the corresponding author on reasonable request.

## Abbreviations

FMNL: Formin-like
FMNL1: Formin-like protein 1
FMNL2: Formin-like protein 2
FMNL3: Formin-like protein 3
DMEM: Dulbecco’s Modified Eagle’s Medium
RPMI: Roswell Park Memorial Institute
FBS: Fetal bovine serum
siRNA: small interfering RNA
NC siRNA: Negative control siRNA
MTT: 3-(4,5-dimethylthiazol-2-yl)-2,5-diphenyltetrazolium bromide dye
CCK8: Cell counting kit-8
PI: Propidium iodide
SDS-PAGE: Sodium dodecyl sulfate-polyacrylamide gel electrophoresis
PVDF: Polyvinylidene difluoride
TIMER: Tumor immune estimation resource
TCGA: The cancer genome atlas
SD: Standard deviation
CDKs: Cyclin-dependent kinases
CKIs: Cyclin-dependent kinase inhibitors
Rb: Retinoblastoma protein
DAPI: Phenylindole
BSA: Bovine serum albumin
