# Supplementary material for: Formin-like protein 2 promotes cell proliferation by a p27-related mechanism in human breast cancer cells
Source: BMC Cancer. 2021 Jun 30;21:760. doi: 10.1186/s12885-021-08533-w (PMC8247103; doi:10.1186/s12885-021-08533-w)
Supplement: Supplementary file 1 — Additional file 1. [file 12885_2021_8533_MOESM1_ESM.docx]

Below you will see the blot images:


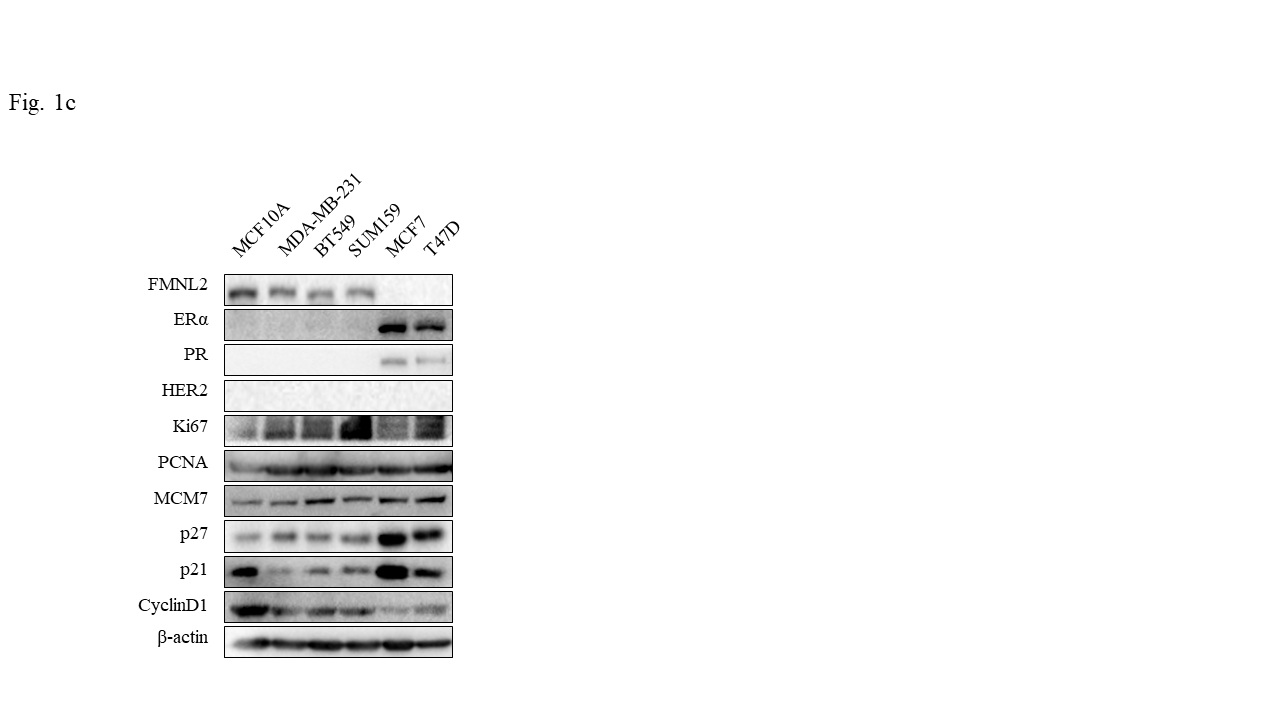


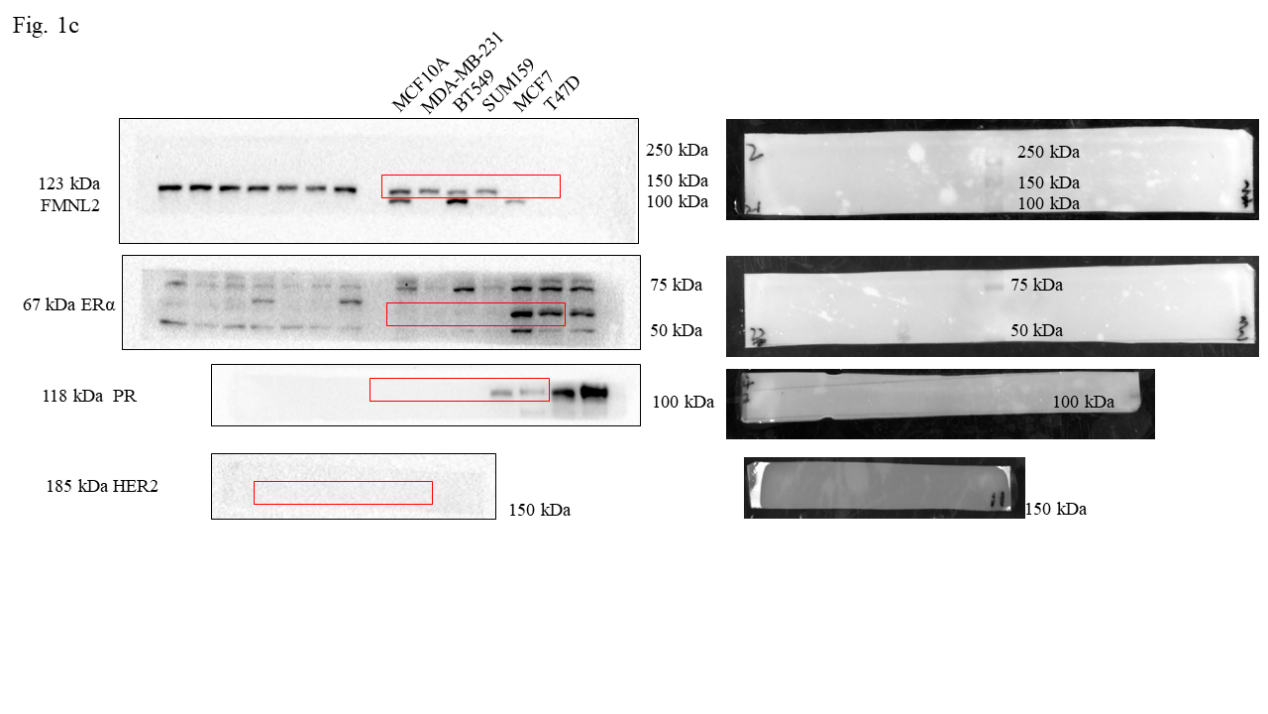

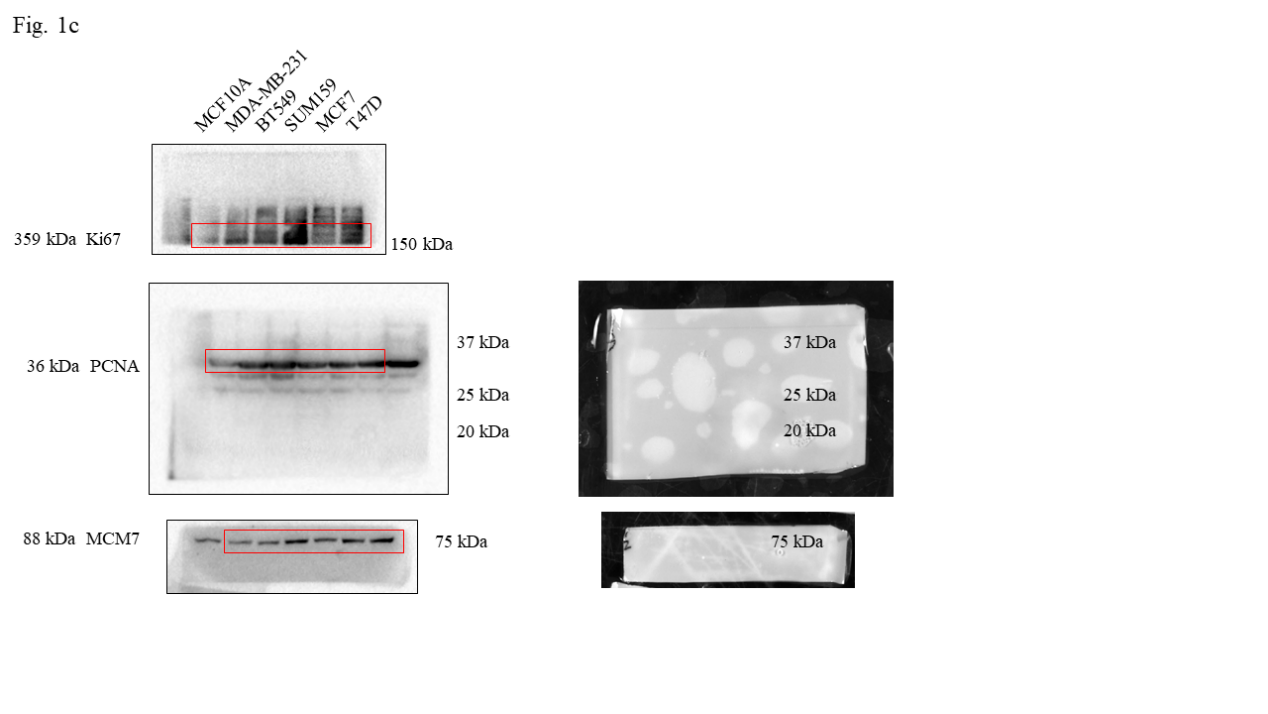


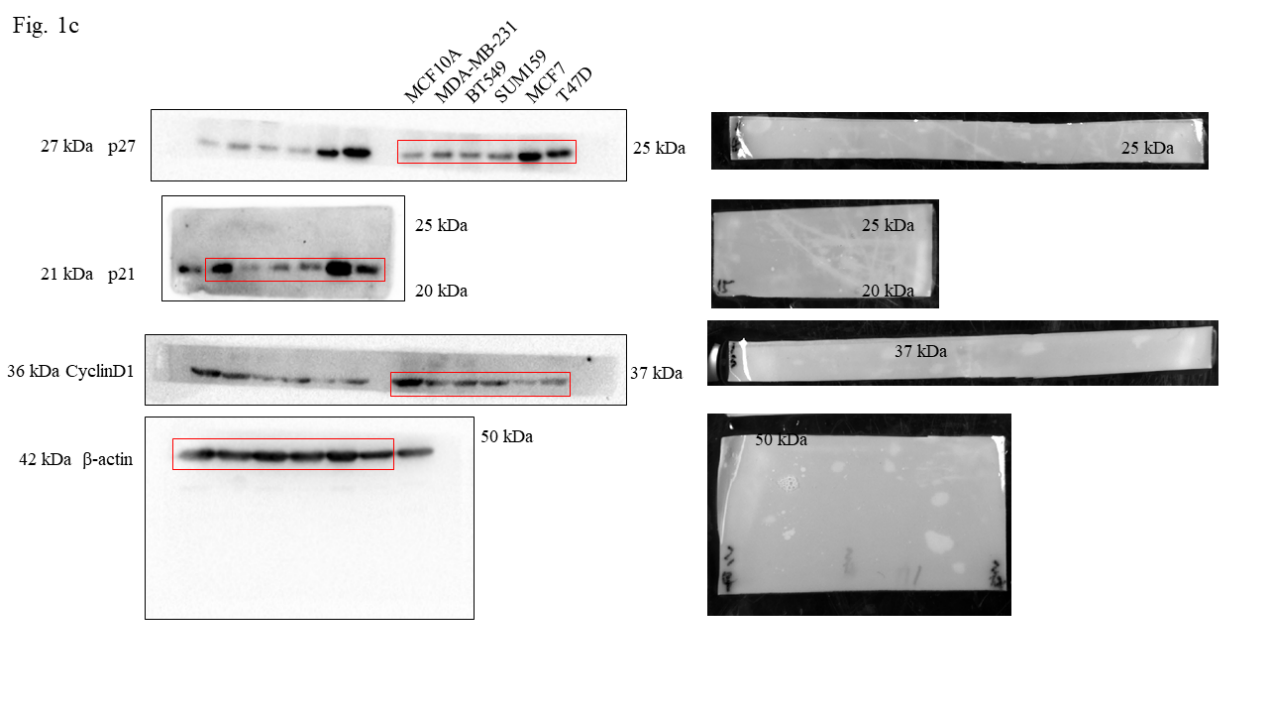


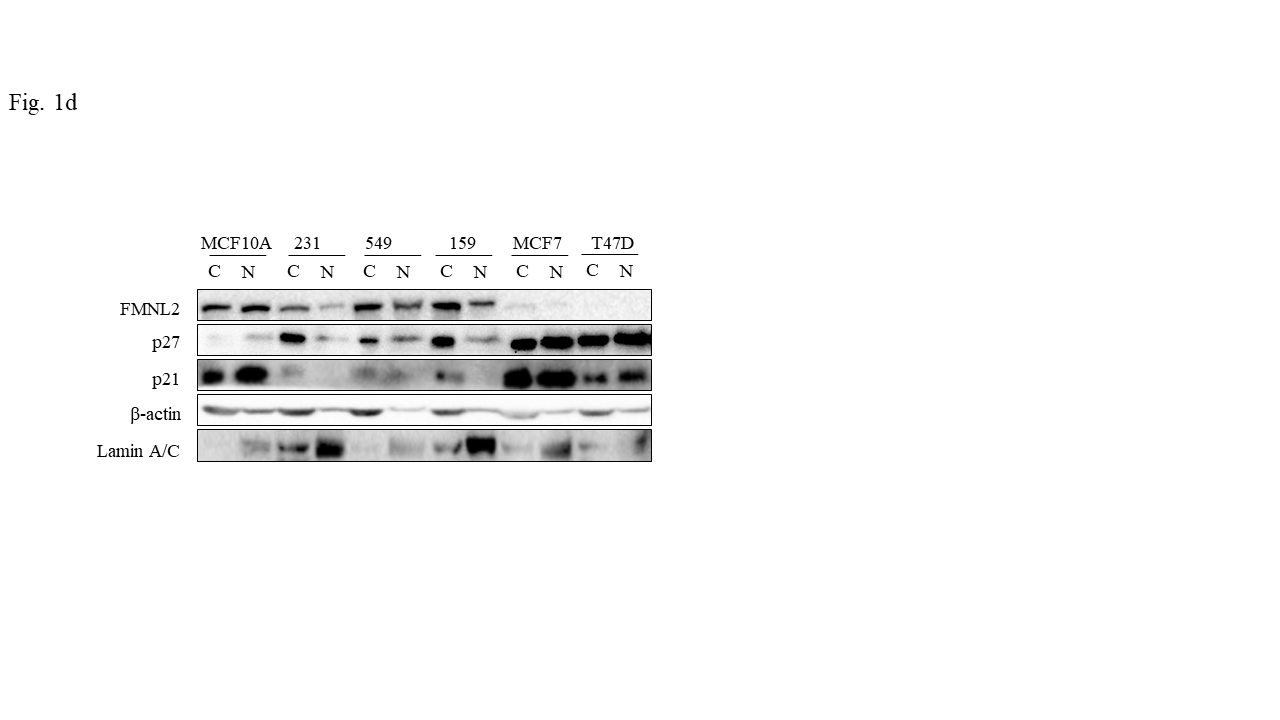

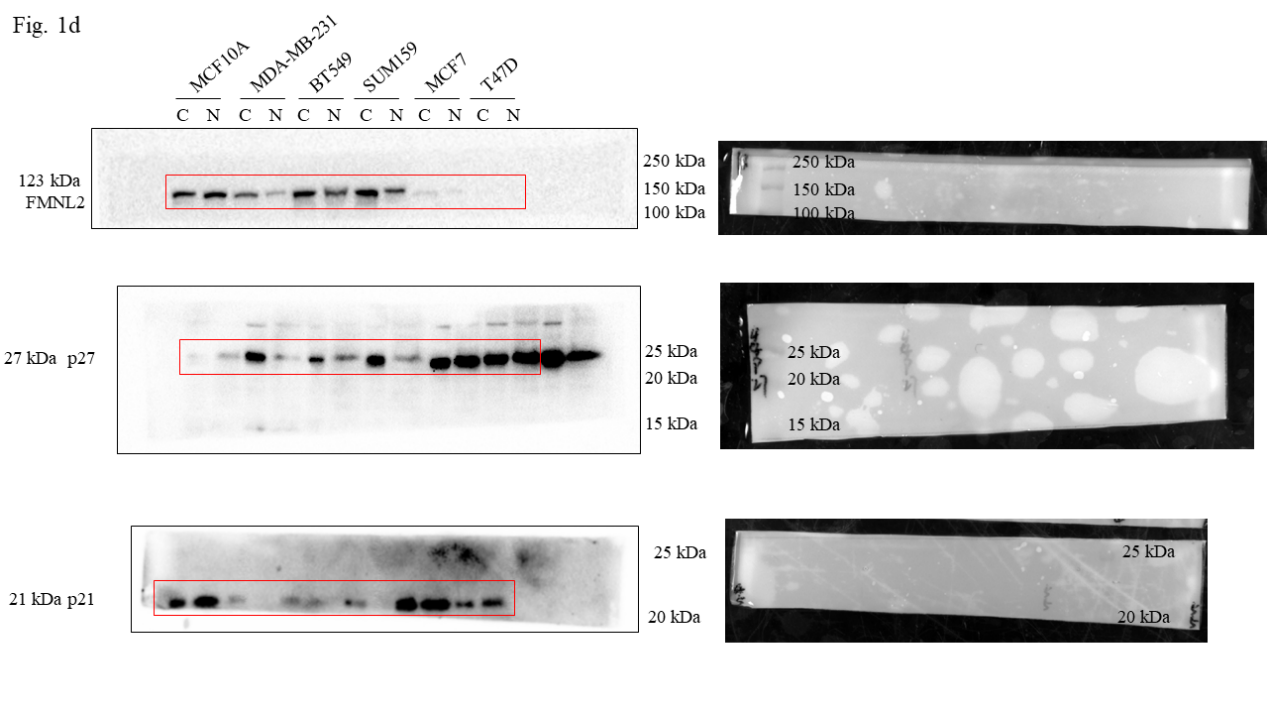

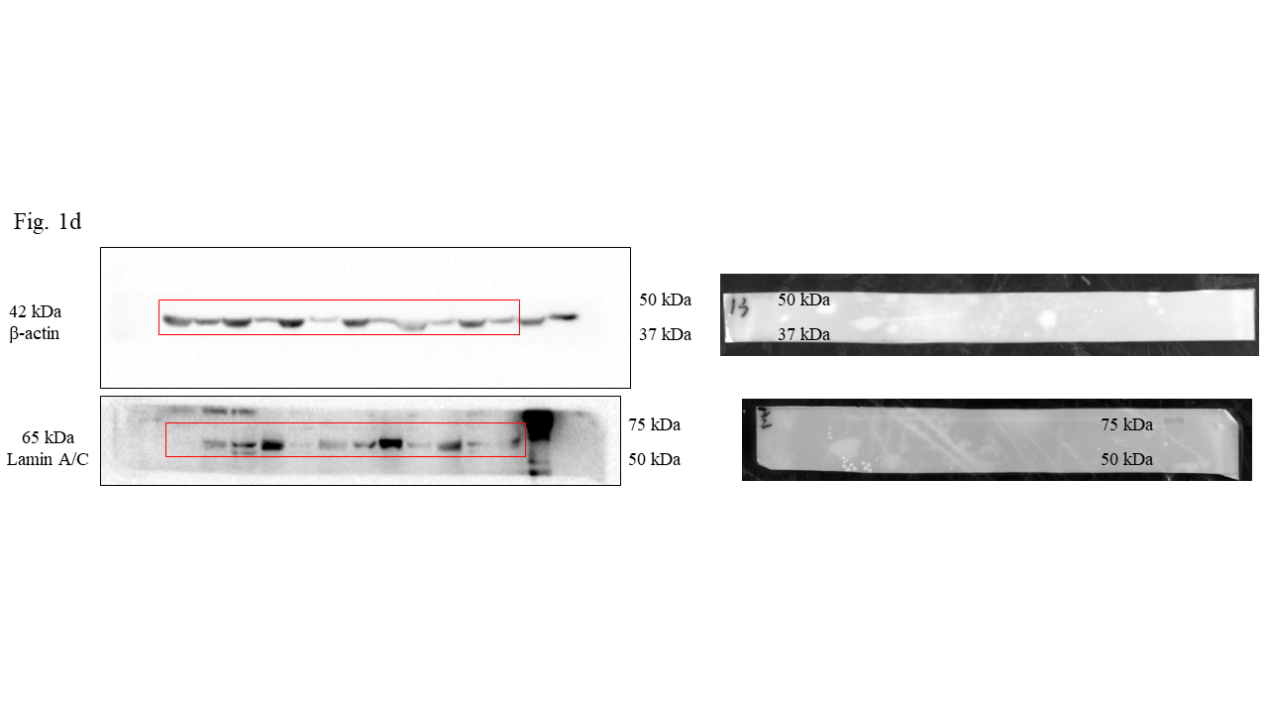


**Fig. 1** The correlation of FMNL2 with cell proliferation in breast cancer. **a** Association of FMNL2 and Ki67 in tumor tissues from breast cancer patients. **b** The correlation of FMNL2 and Ki67 expression was determined in breast cancer samples using TIMER. **c** and **d** The levels of ERα, PR, HER2, Ki67, PCNA, MCM7, FMNL2, p27, p21, CyclinD1, Lamin A/C, and β-actin proteins were determined by western blotting and representative blots were shown. *: *P*<0.05 *vs* FMNL2 negative group.


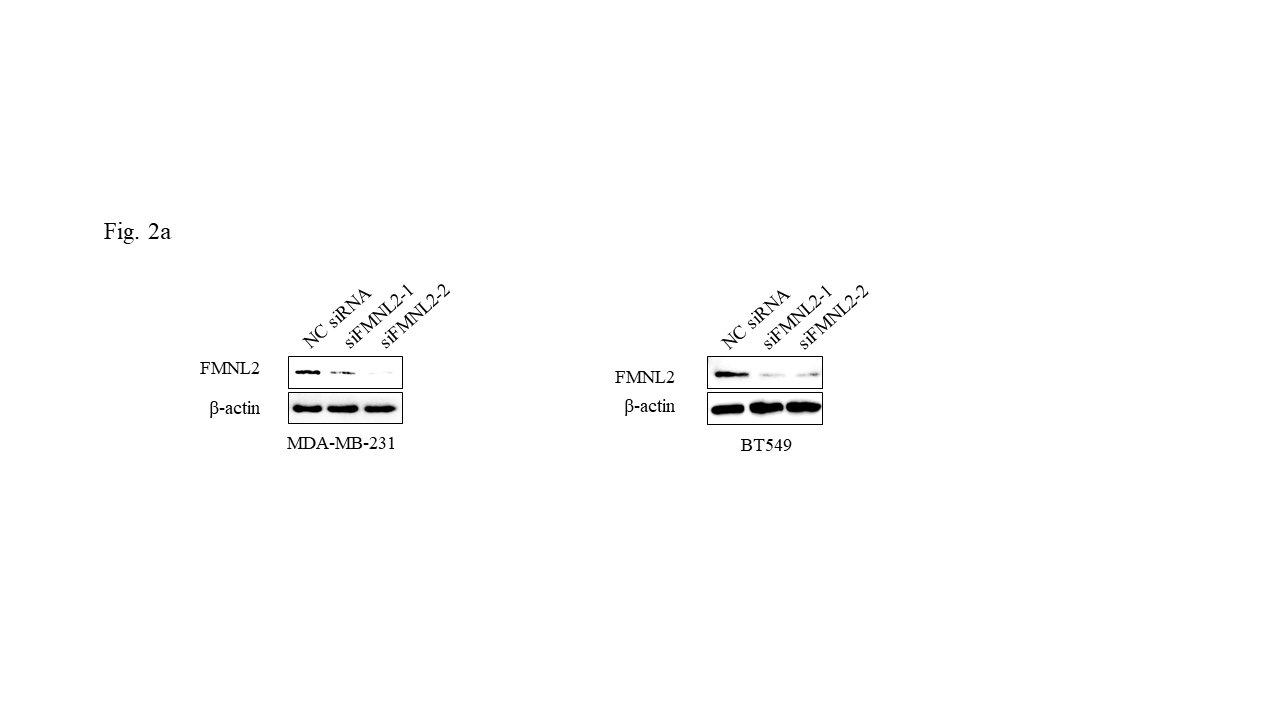


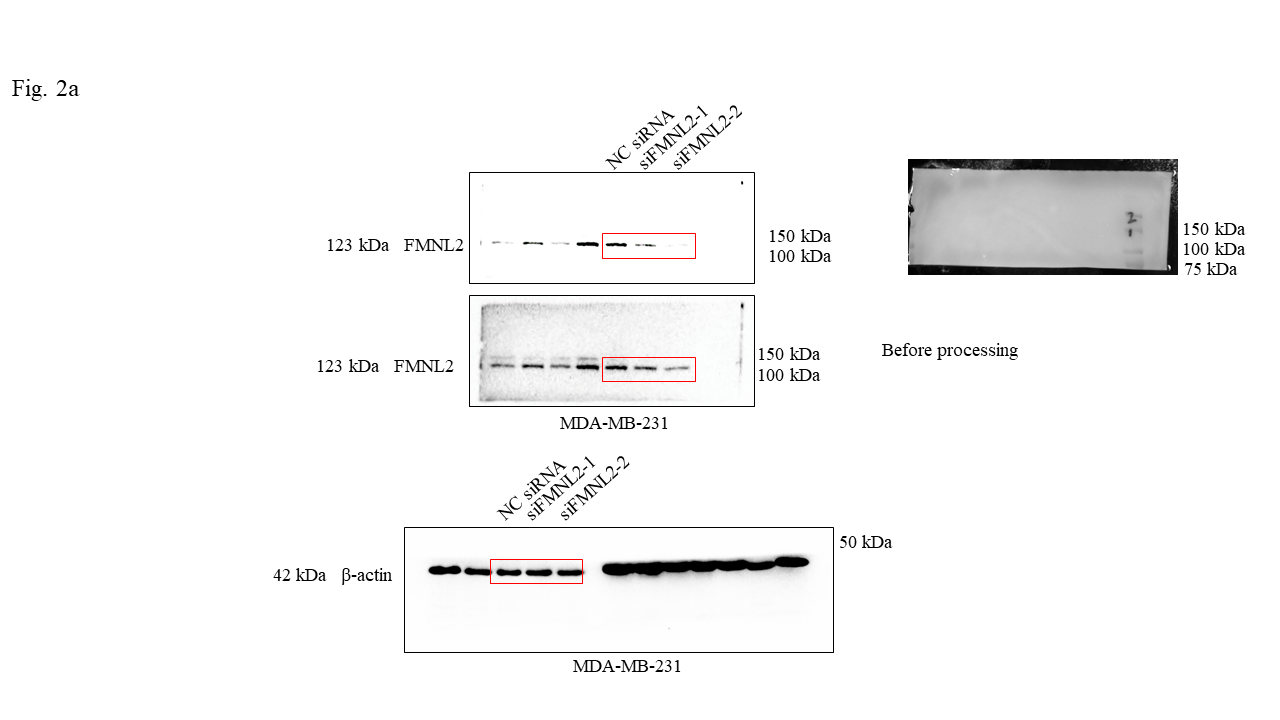


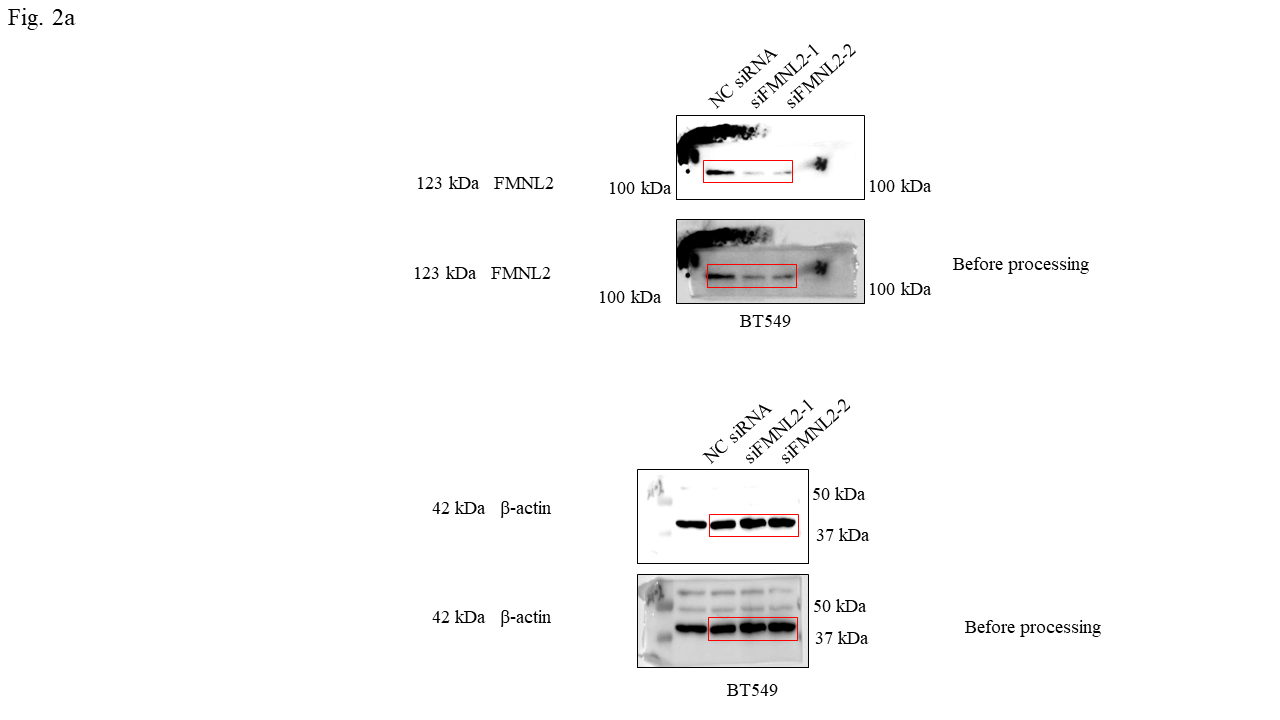


**Fig. 2** Effects of FMNL2 silencing on cell proliferation in MDA-MB-231 and BT549 cells. After transfection for 48 h, cultured cells were processed for indicated assays. **a** The expression of FMNL2 protein was determined by western blotting analyses. **b** The expression of FMNL2 mRNA was determined by qRT-PCR analyses. **c** The cell proliferation was detected by CCK8 assay. **d** The cell viability was examined by MTT assay. **e** The cell number was determined by cell counting assay. **f** The number of forming colonies was counted and analyzed statistically. *: *P*<0.05 *vs* NC siRNA group.


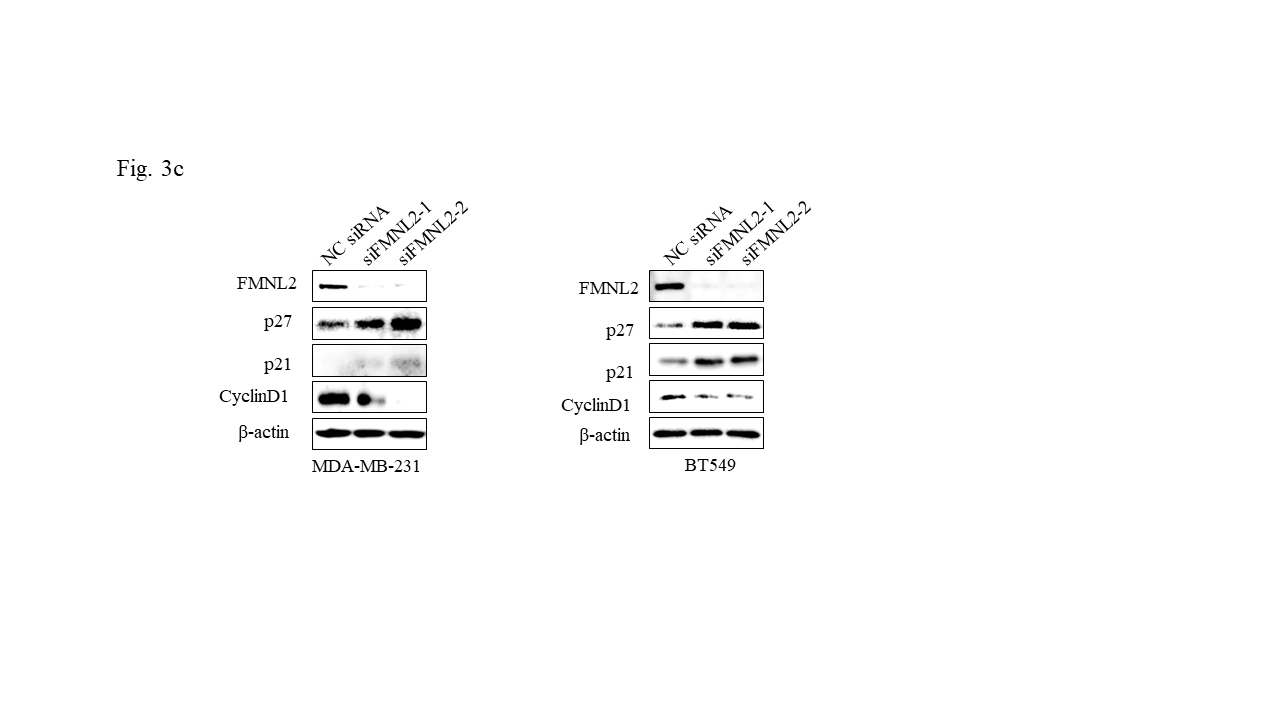


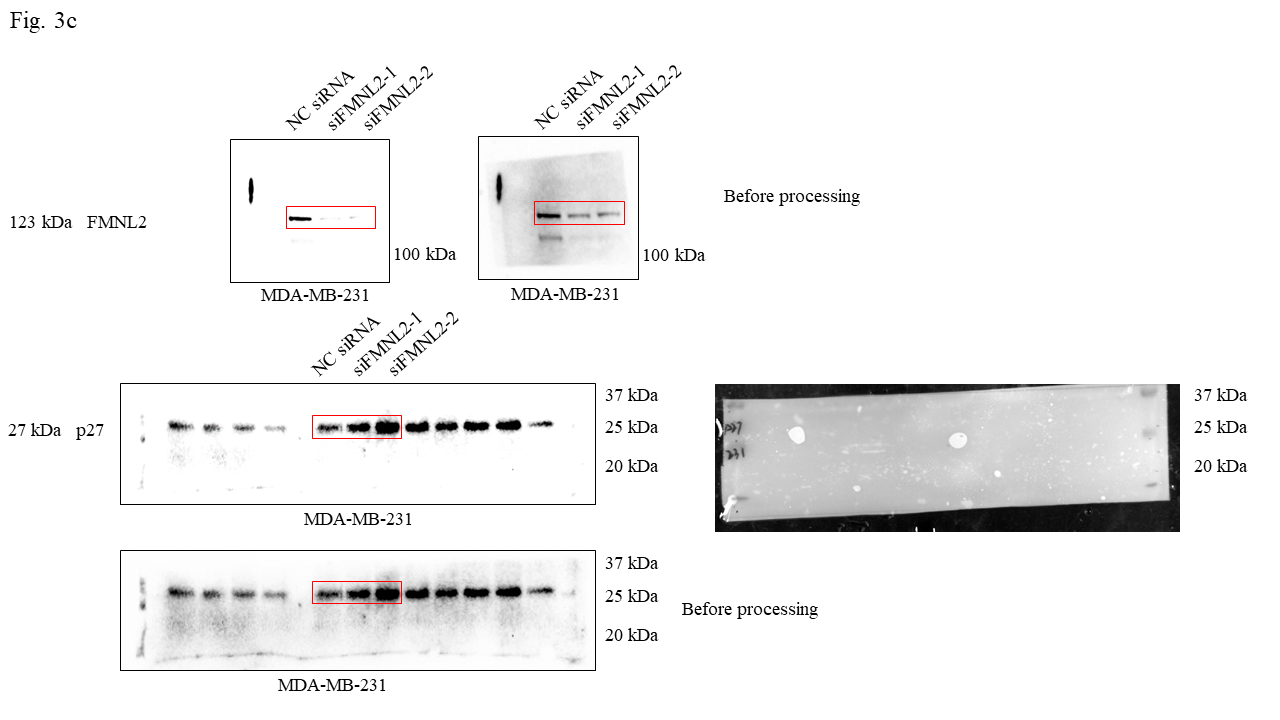


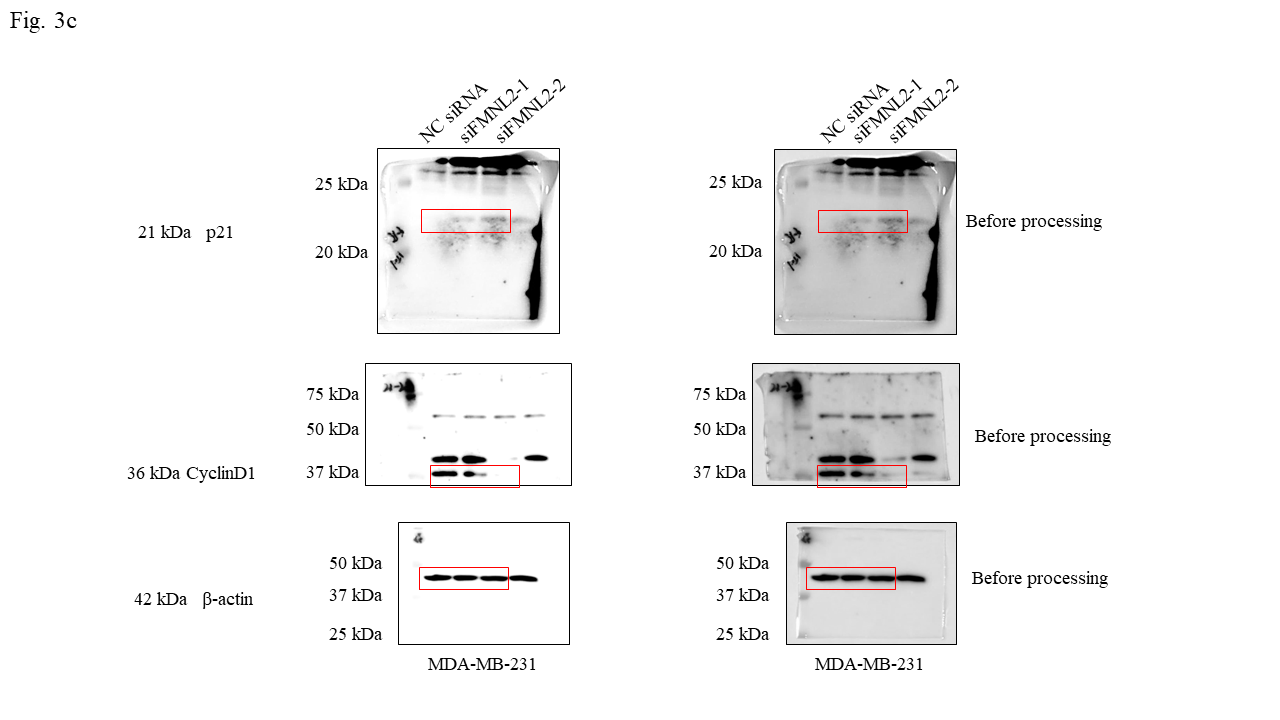


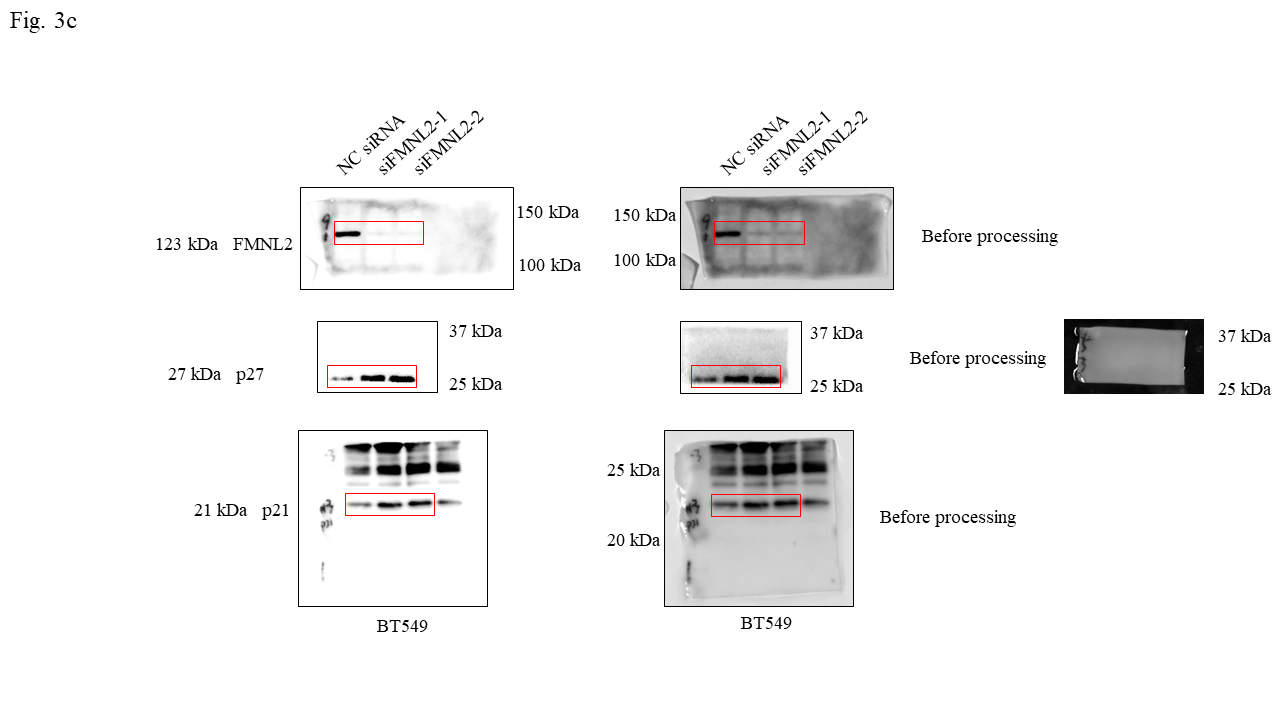


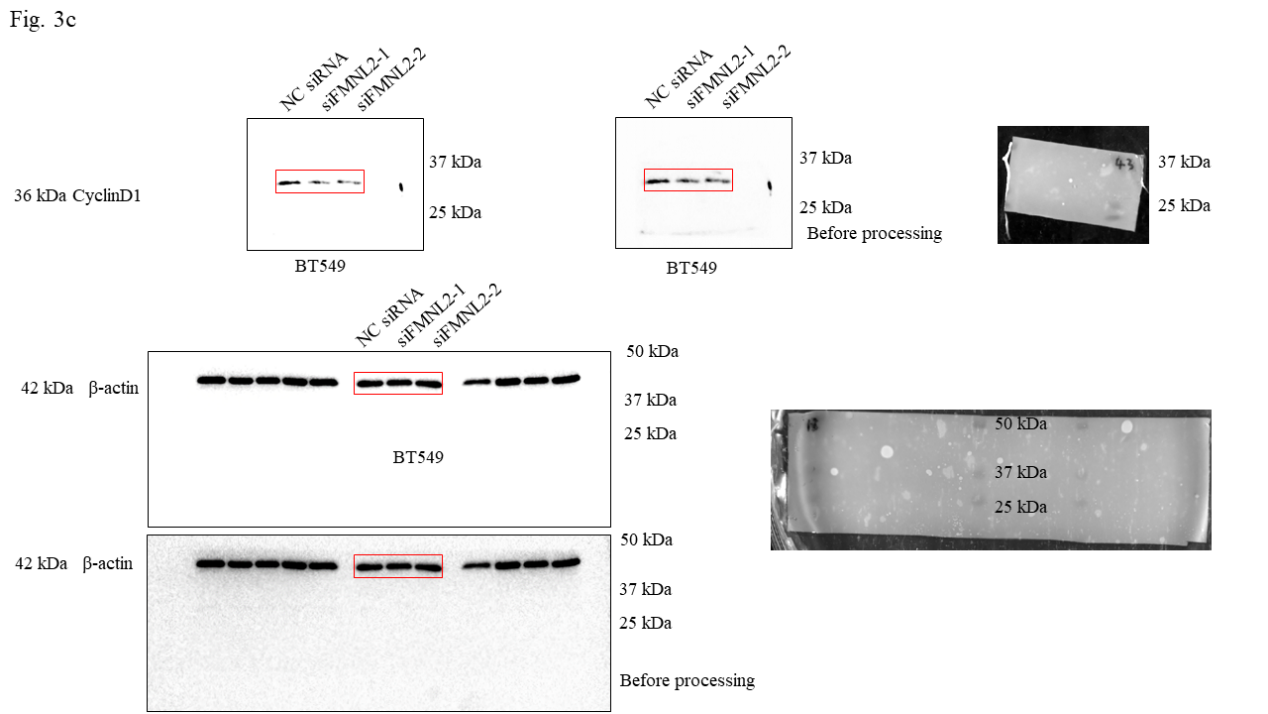


**Fig. 3** Effects of FMNL2 silencing on cell cycle and proliferation markers in MDA-MB-231 and BT549 cells. After transfection for 48 h, cultured cells were processed for indicated assays. a Representative EdU staining images of cells after transfection were shown. b Representative percentage of cells in the G0/G1, G2/M and S phases was detected by ﬂow cytometric analysis. c The levels of FMNL2, p27, p21, CyclinD1, and β-actin proteins were determined by western blotting and representative blots were shown. d The levels of CDK4 and CDK6 mRNA were determined by qRT-PCR. e The CDK4/CyclinD1 kinase activity was determined. Scale bar, 50 μm. *: P<0.05 vs NC siRNA group.


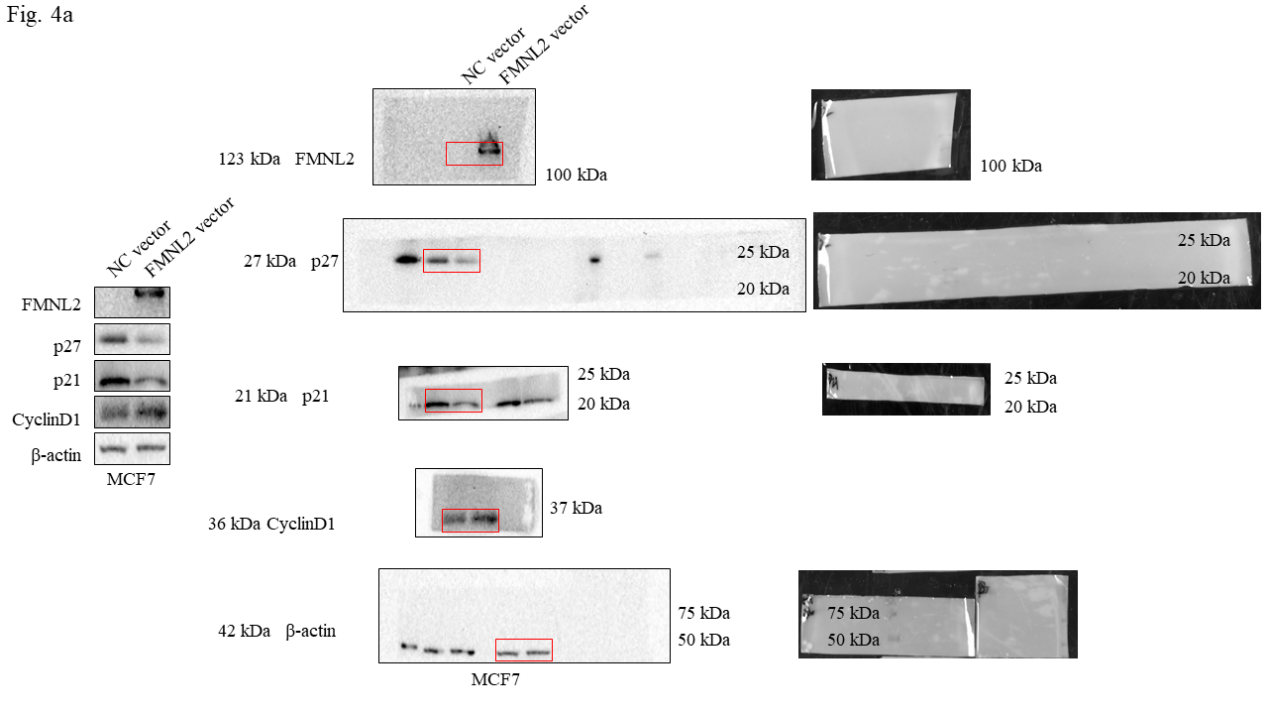


**Fig. 4** Effects of FMNL2 overexpression on cell proliferation in MCF7 cells. After transfection for 48 h, cultured cells were processed for indicated assays. **a** The levels of FMNL2, p27, p21, CyclinD1, and β-actin proteins were determined by western blotting and representative blots were shown. **b** The cell viability was examined by MTT assay. **c** The number of forming colonies was counted and analyzed statistically. **d** Representative percentage of cells in the G0/G1, G2/M and S phases was detected by ﬂow cytometric analysis. **e** The CDK4/CyclinD1 kinase activity was determined. *: *P*<0.05 *vs* NC vector group.


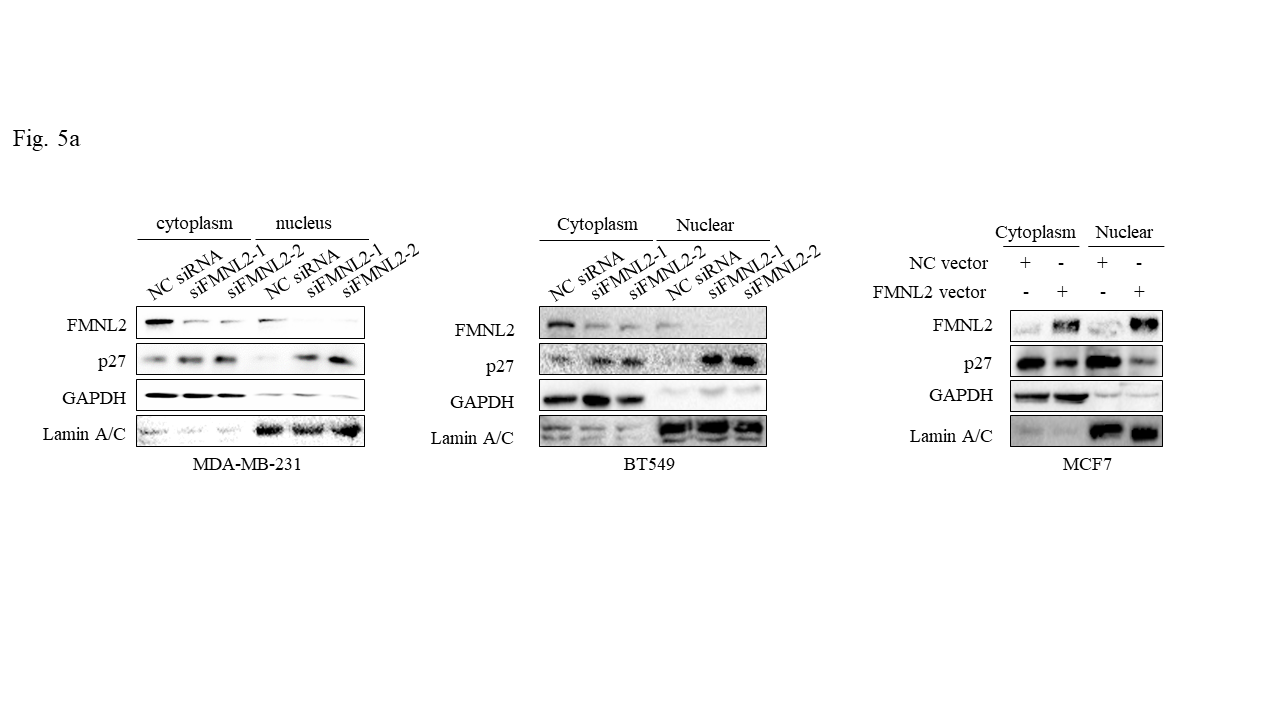


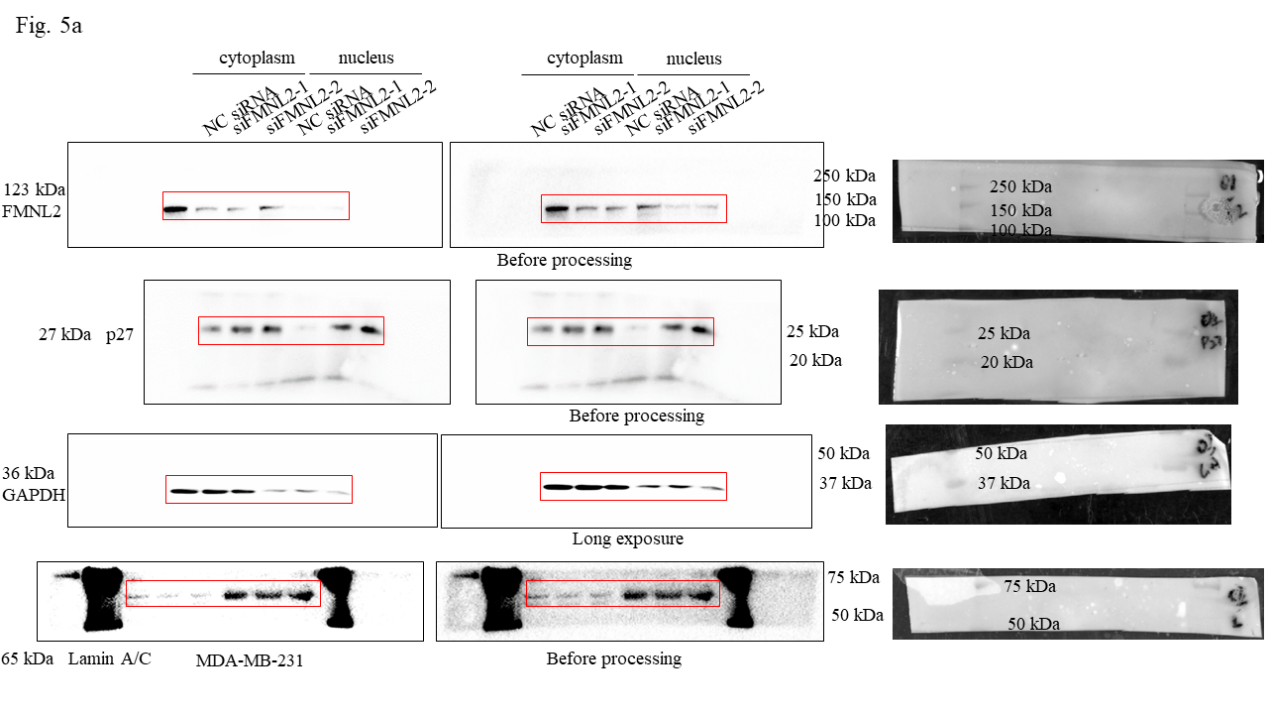


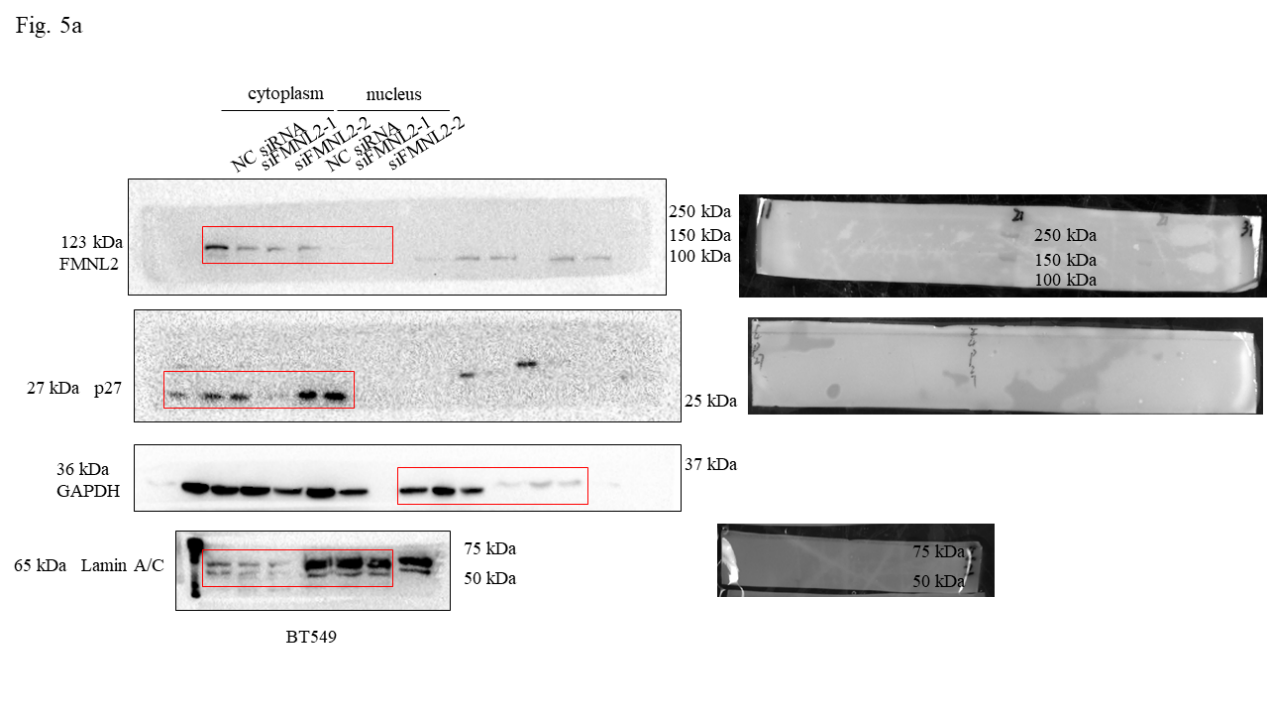


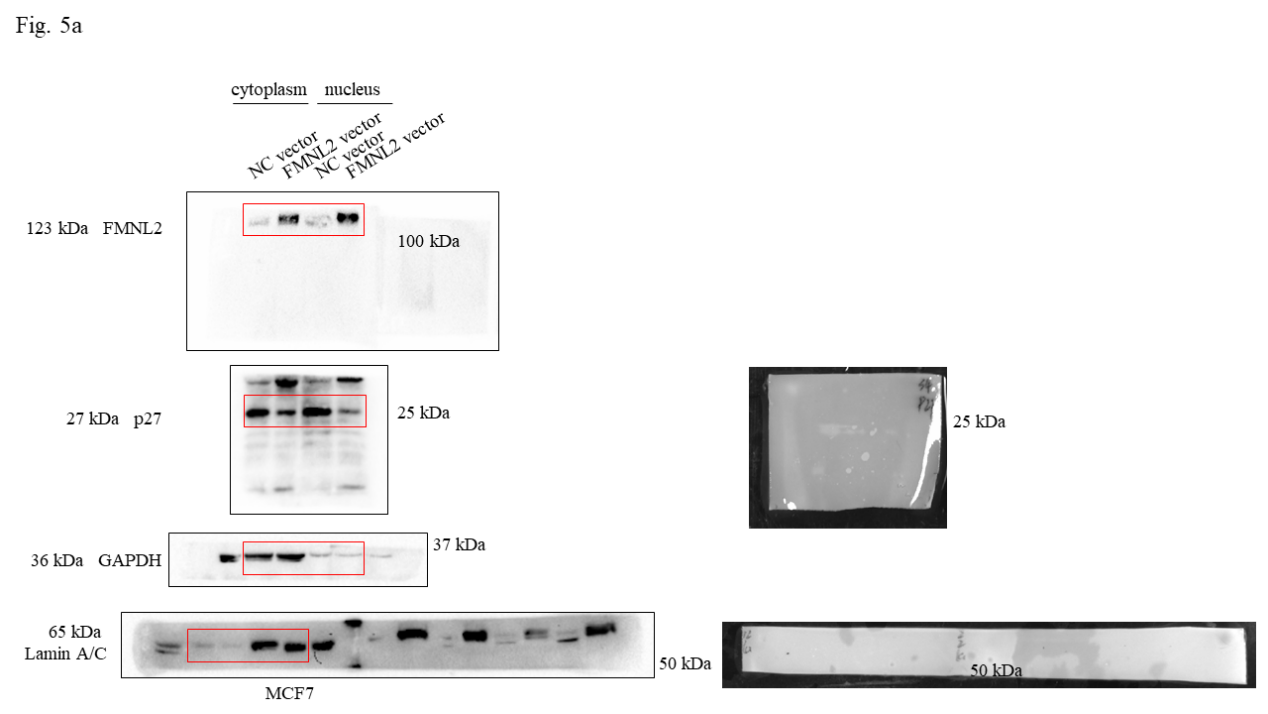


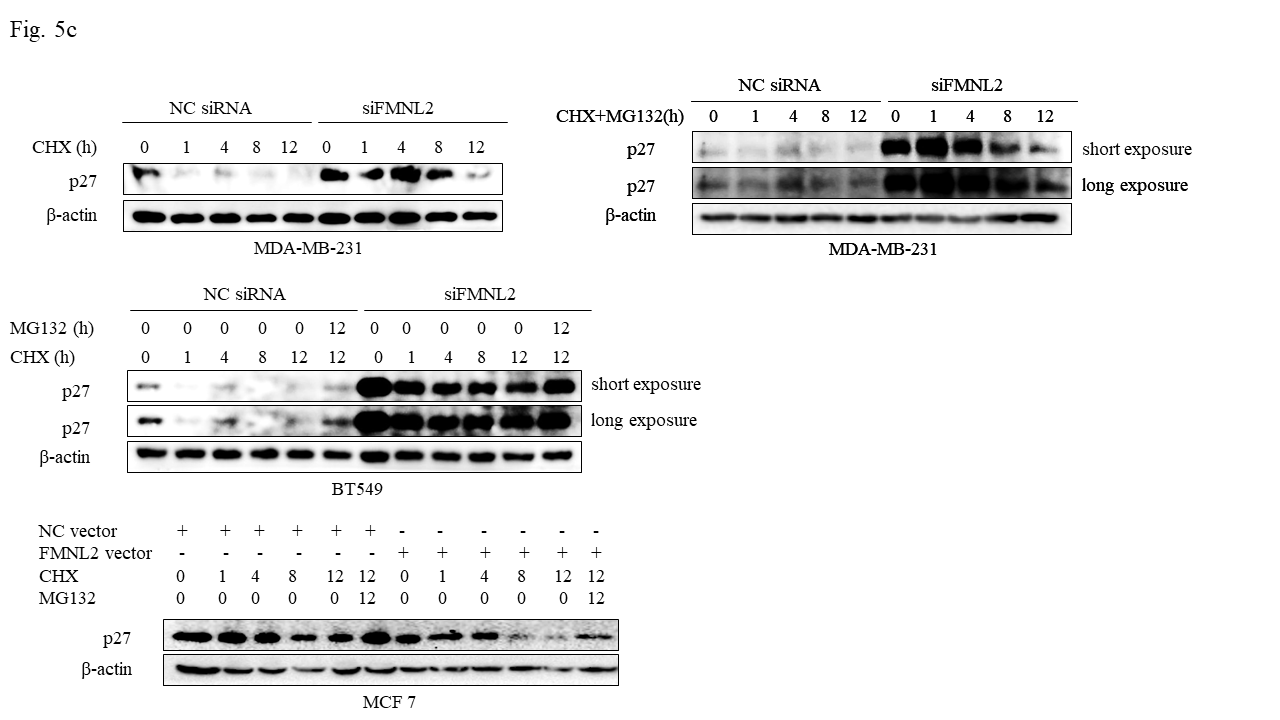


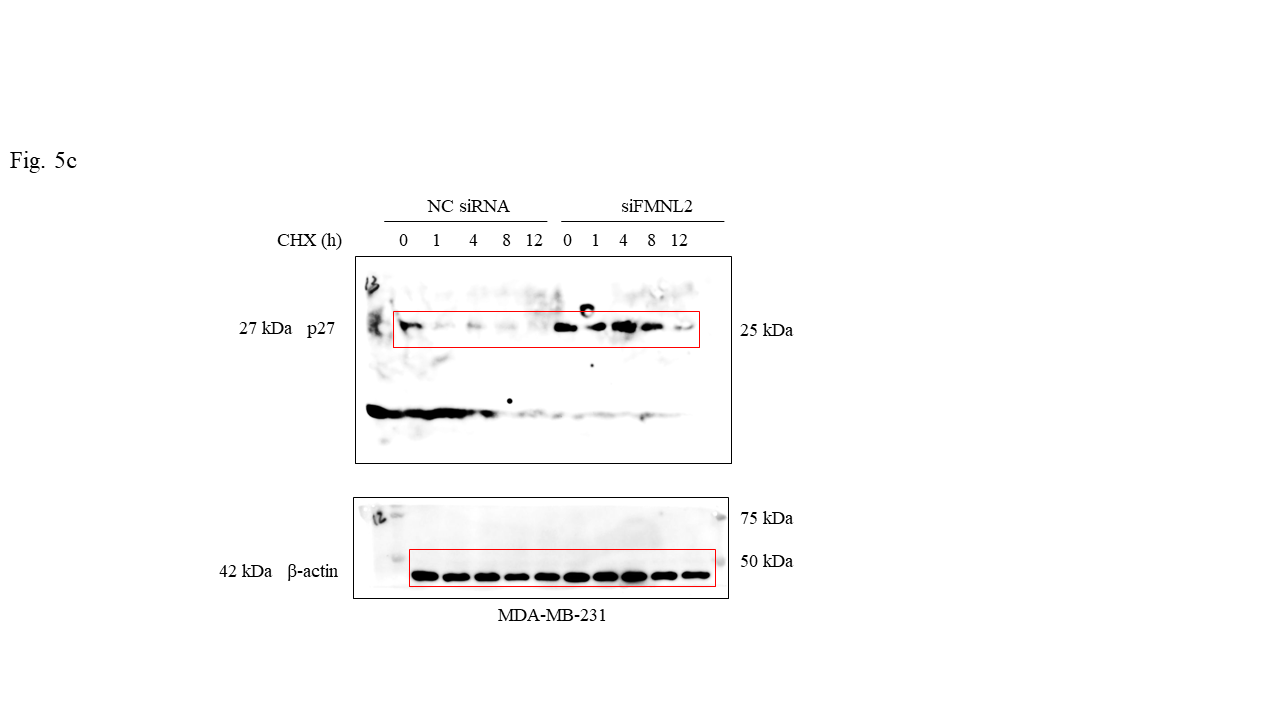


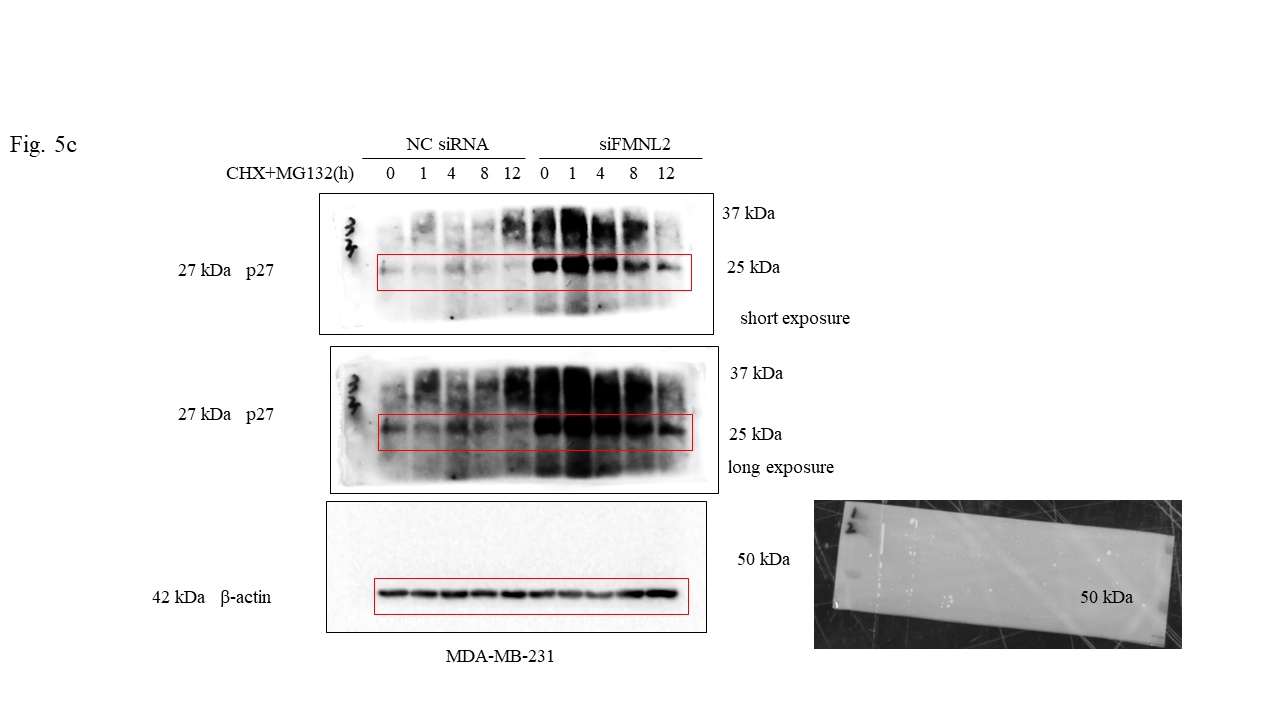


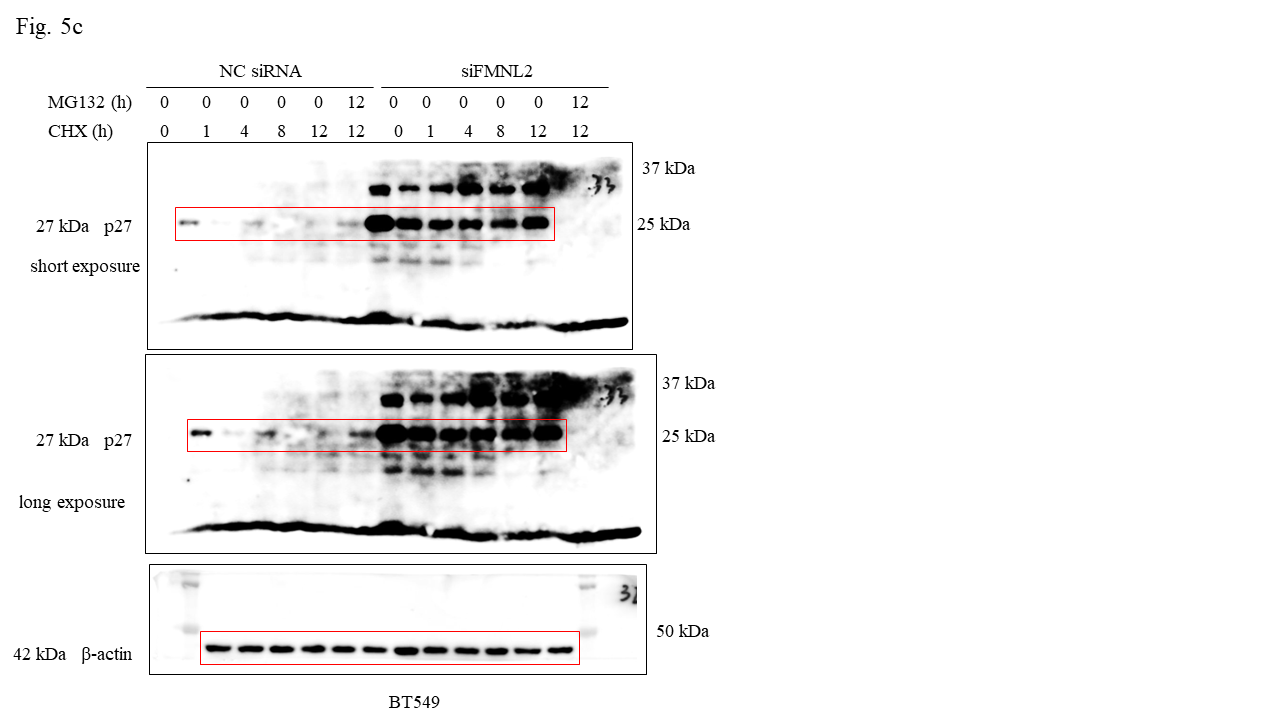


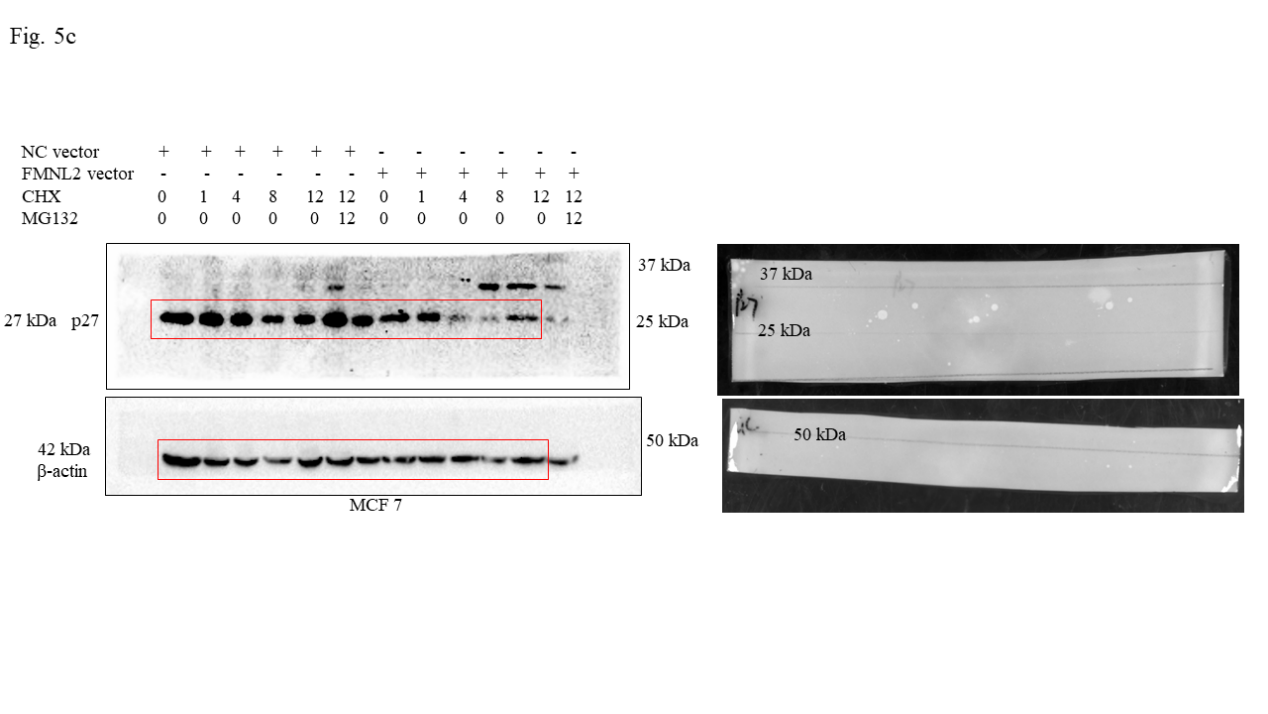


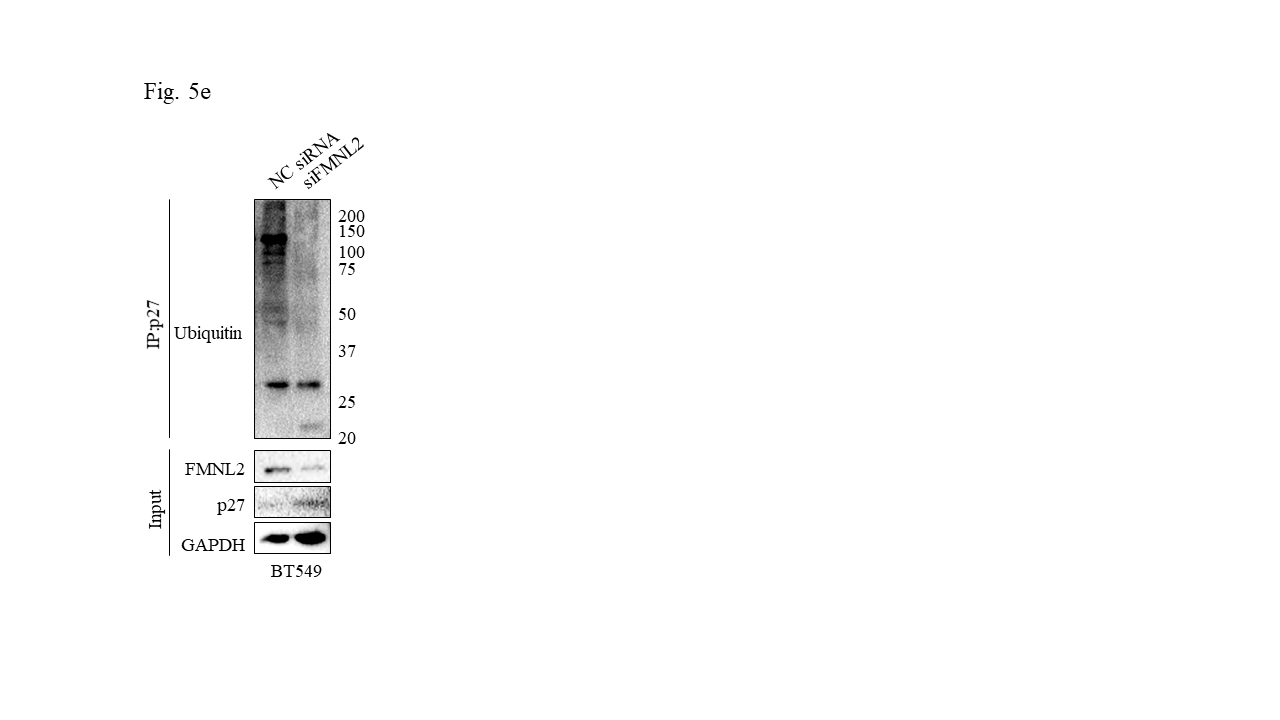


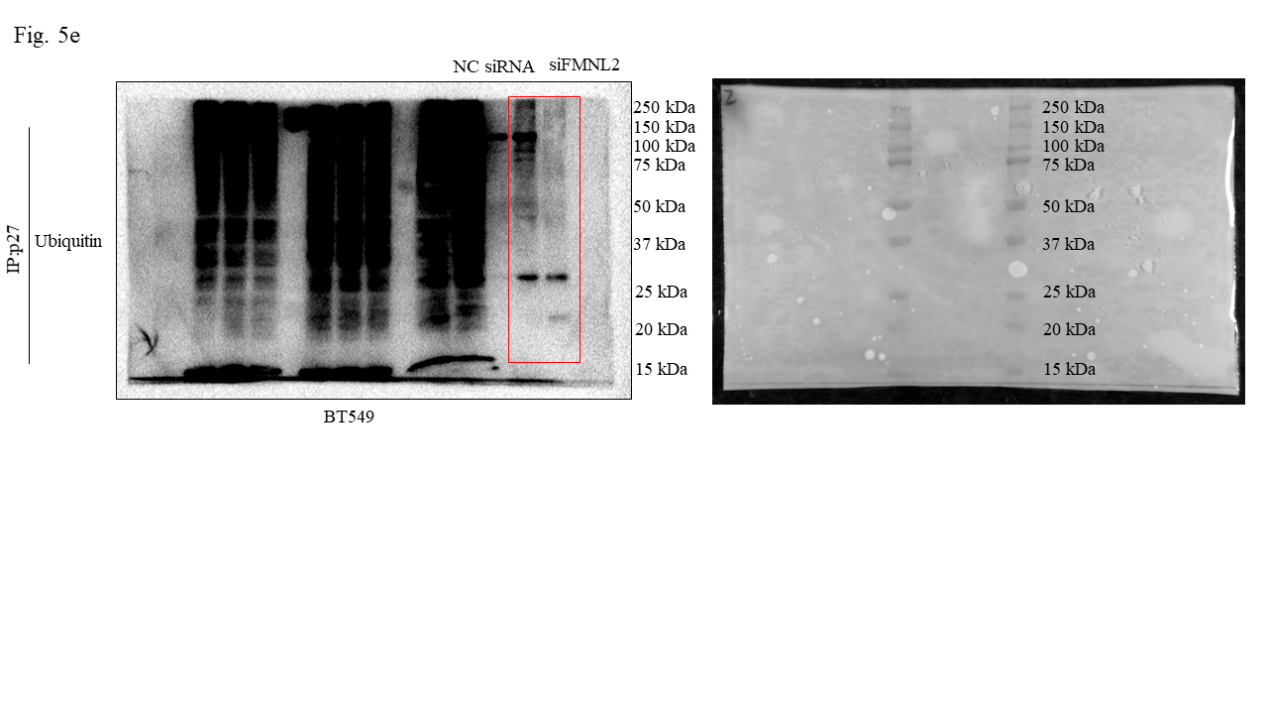


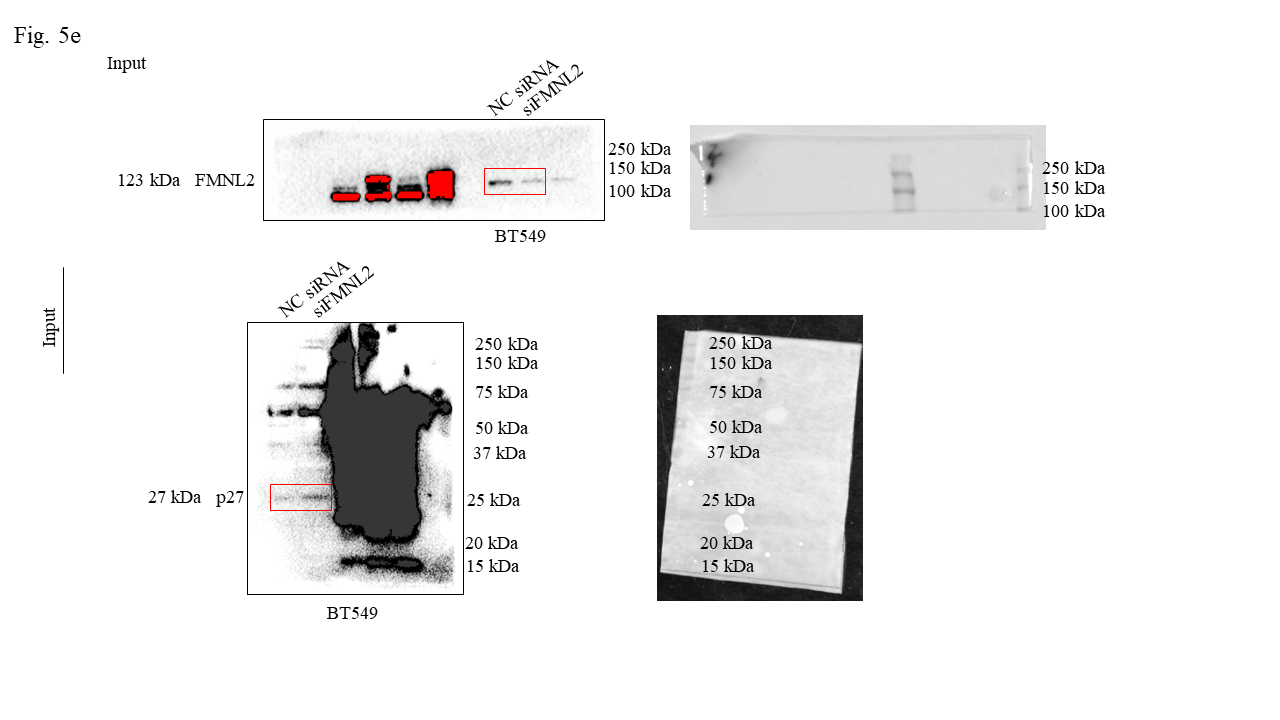


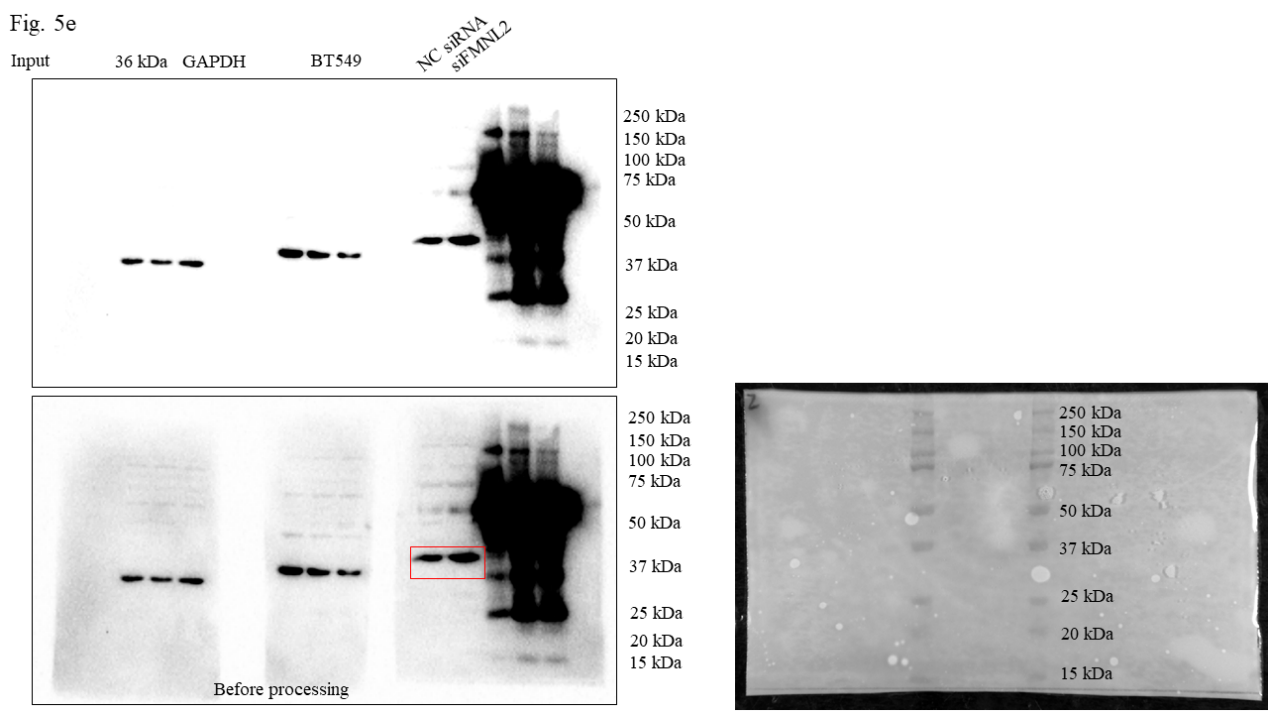


**Fig.5** Effects of FMNL2 on the nuclear levels of p27 and the degradation of p27. **a** Cellular fractionation experiments were performed to examine the levels of FMNL2 and p27 protein in the nucleus and cytoplasm. GAPDH and Lamin A/C were used as loading controls. **b** Representative fluorescent images of p27 were displayed. **c** After transfection for 48 h, cultured cells were incubated with cyclohexmide (CHX, 100 μg/mL) or MG132 (5 μM) for the indicated times. The levels of p27 protein were detected by western blotting. **d** A plot of normalized p27 expression is shown. **e** The ubiquitination of p27 was detected by western blotting. Scale bar, 50 μm.


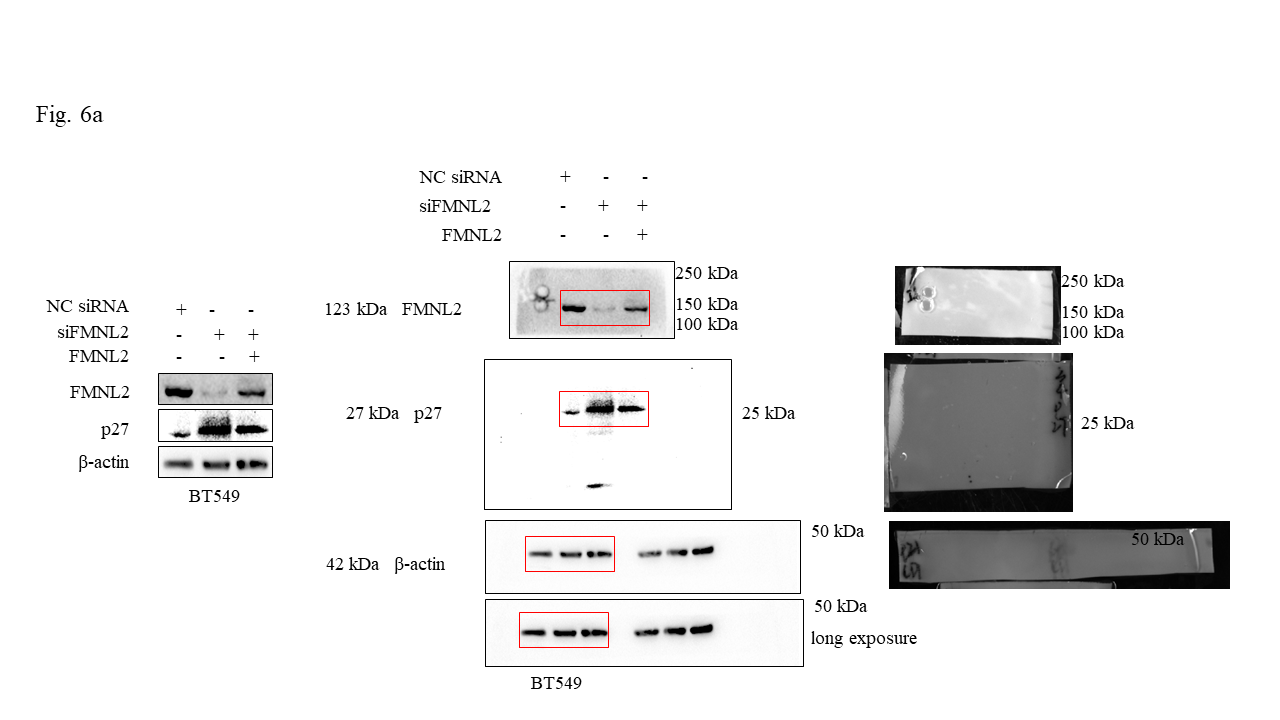


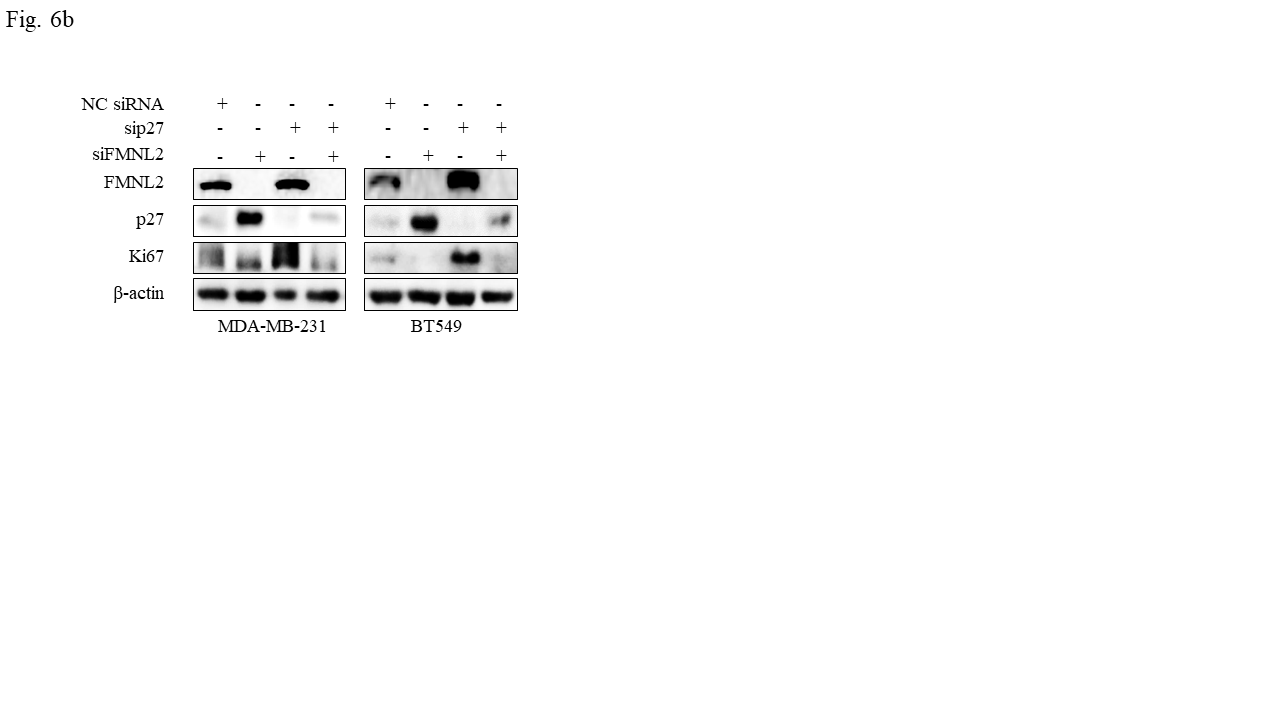


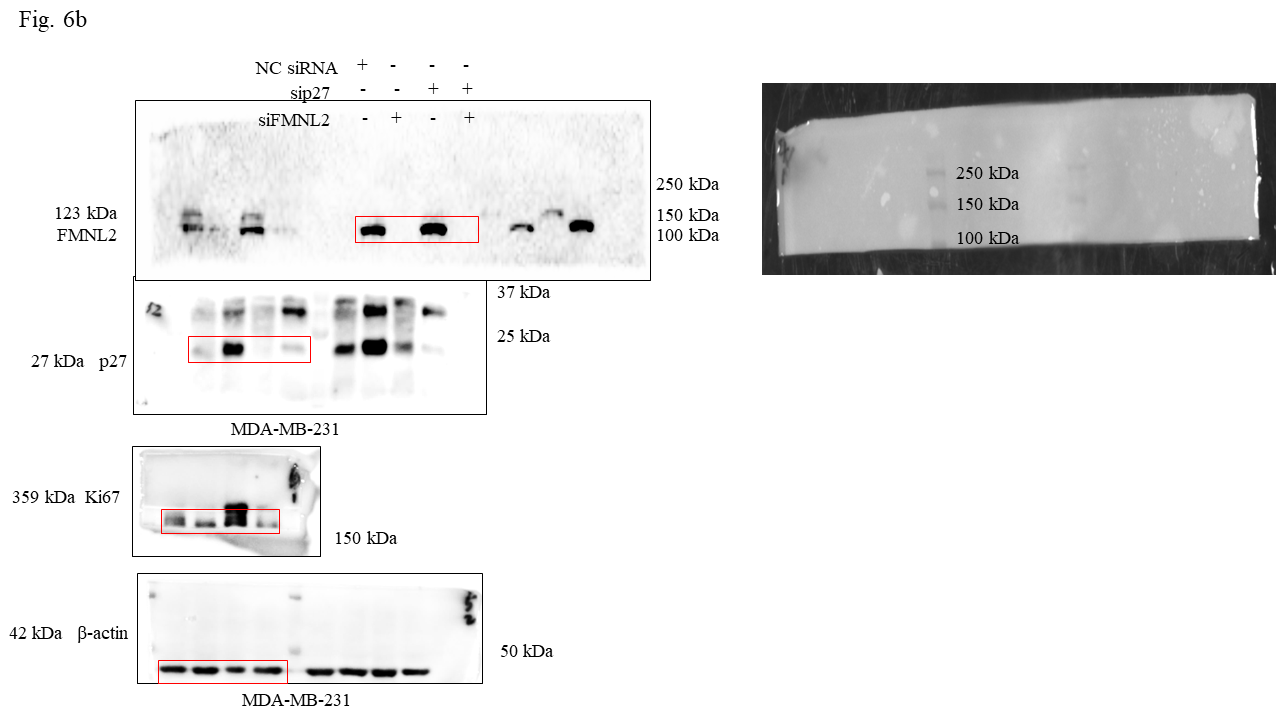


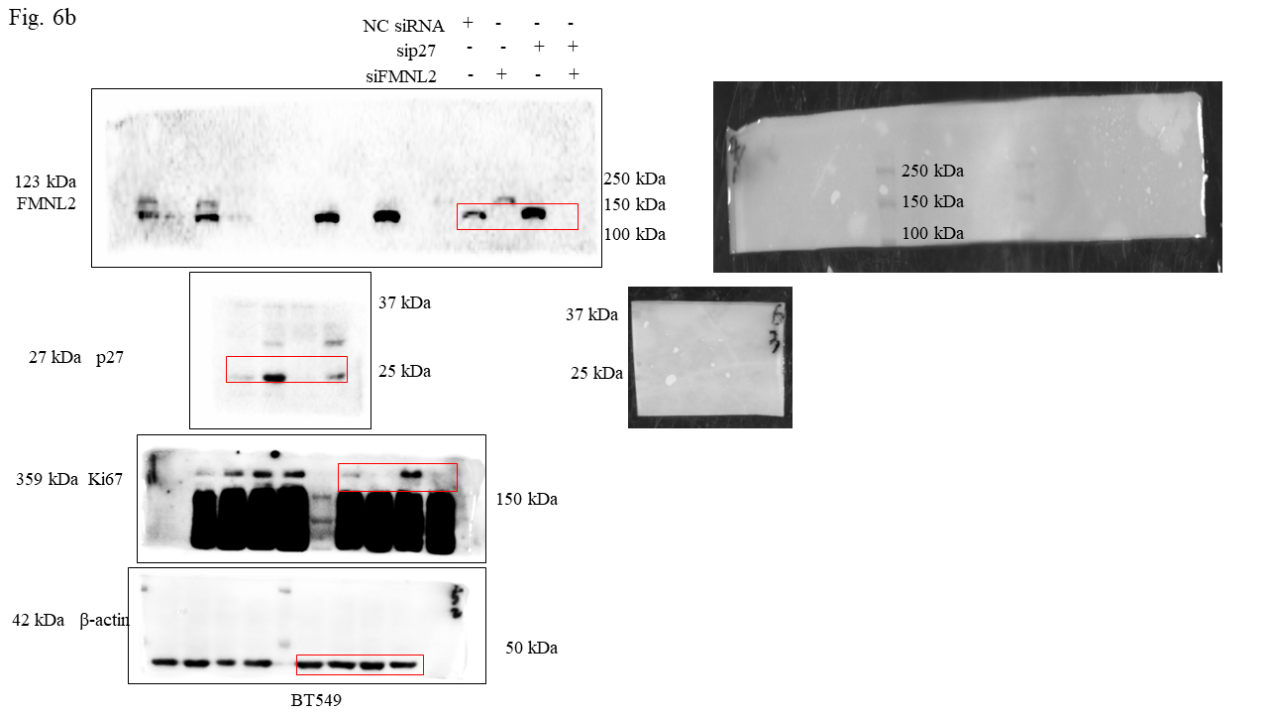


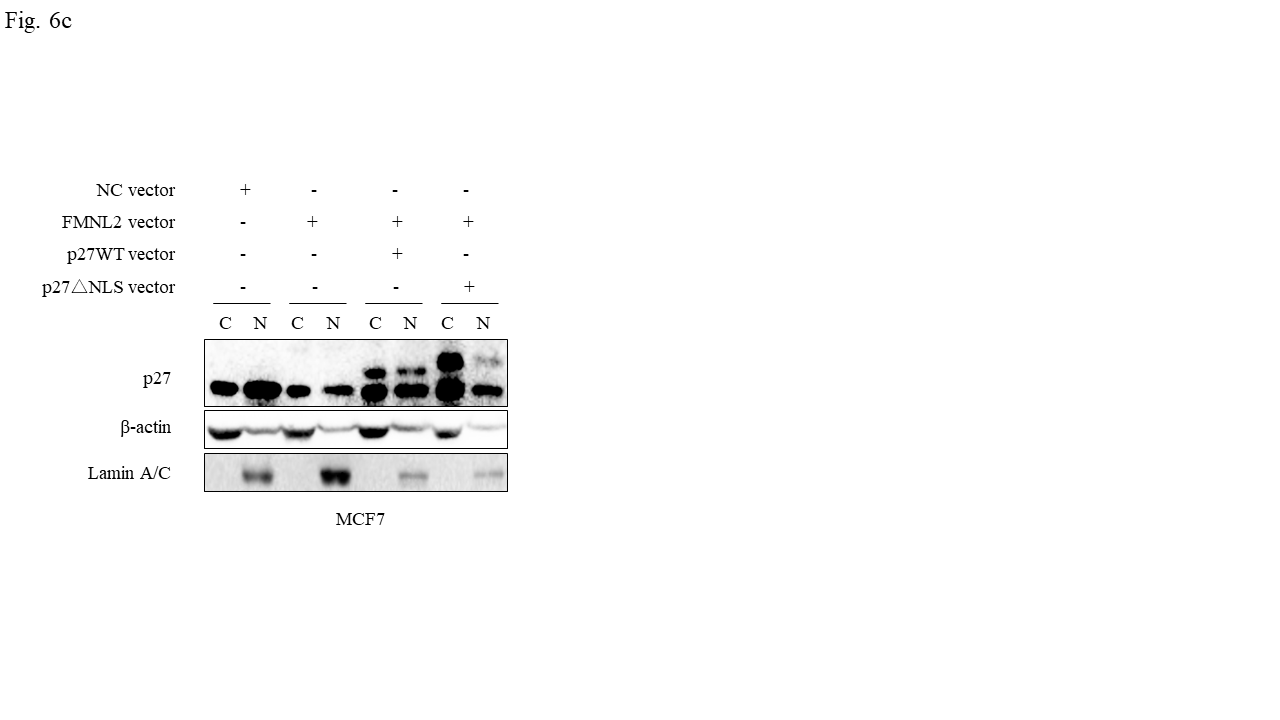


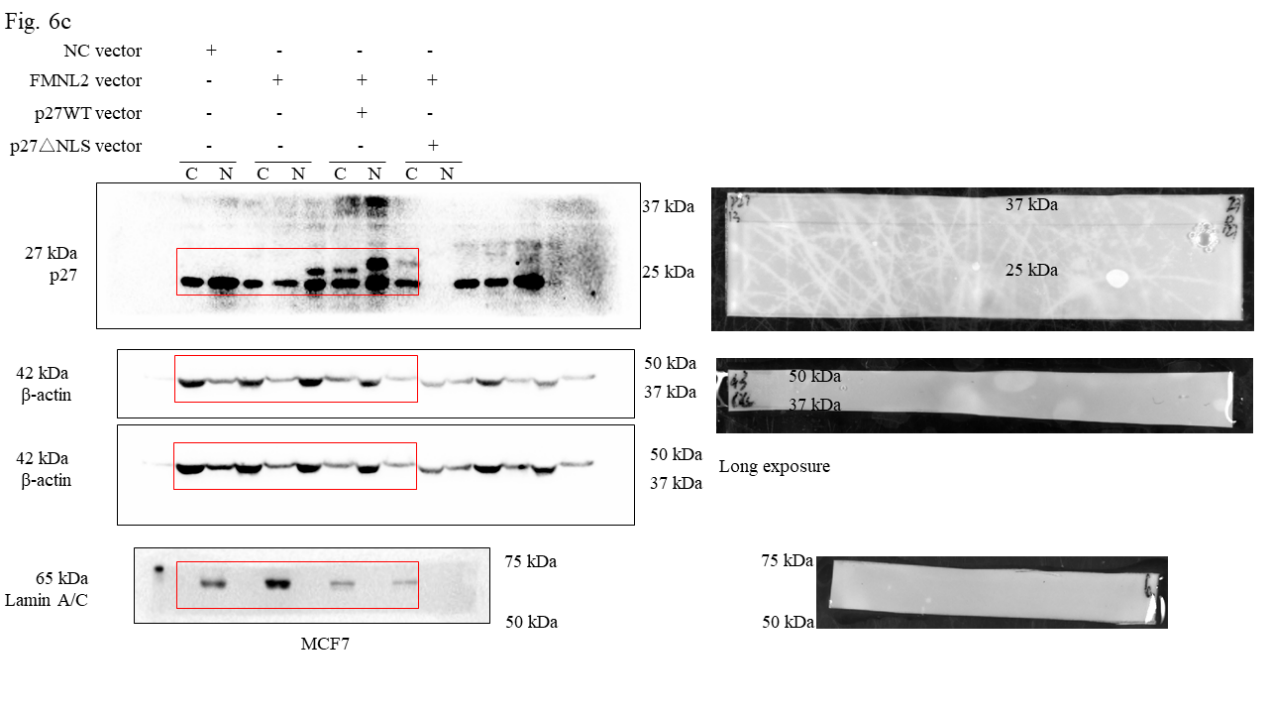


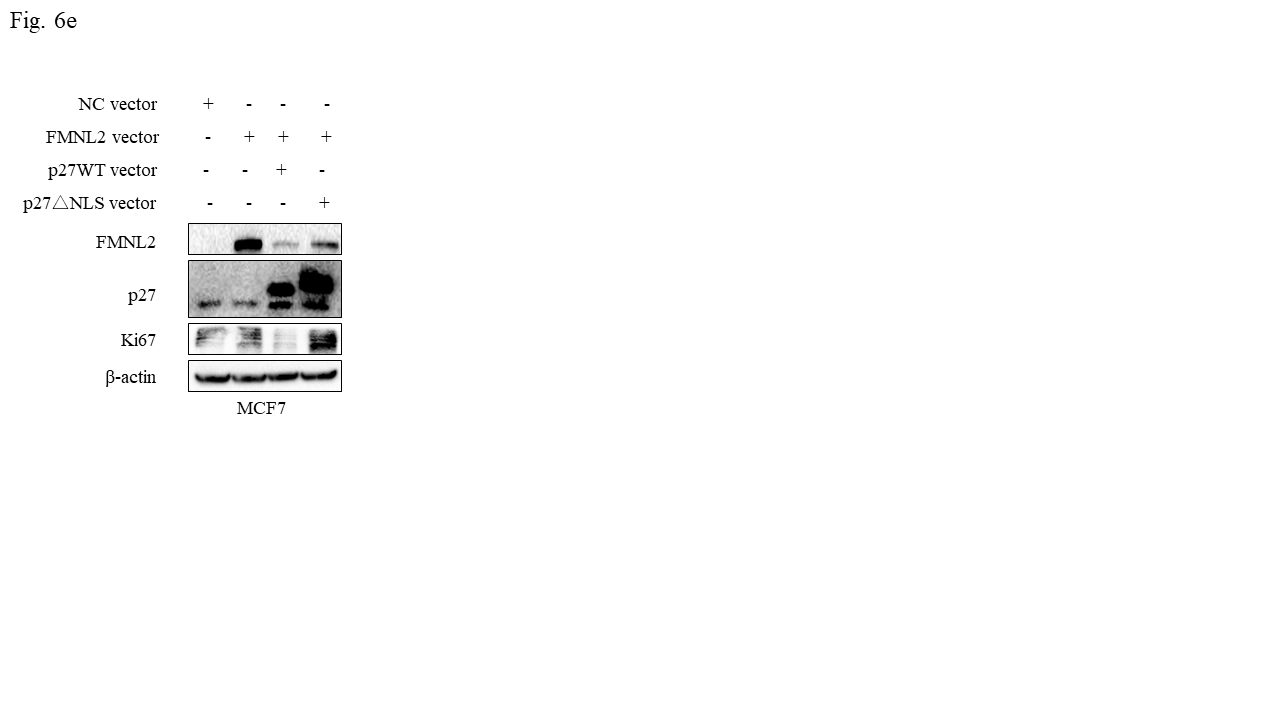


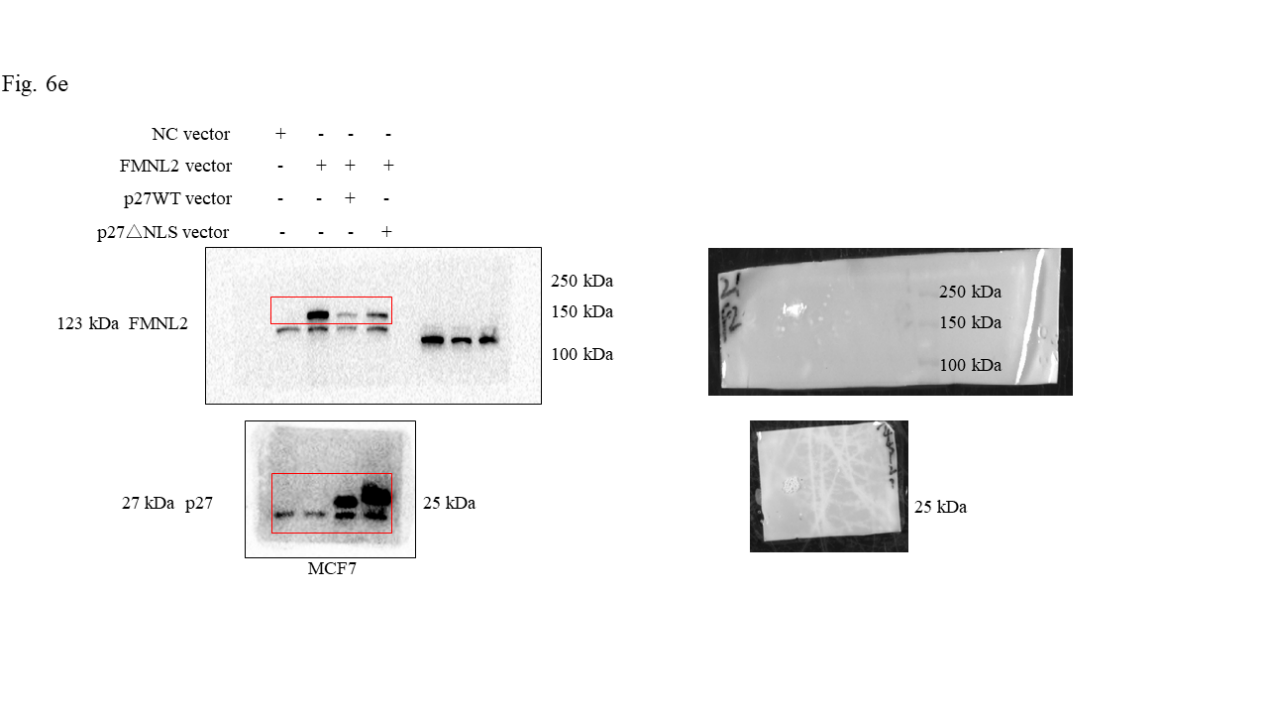


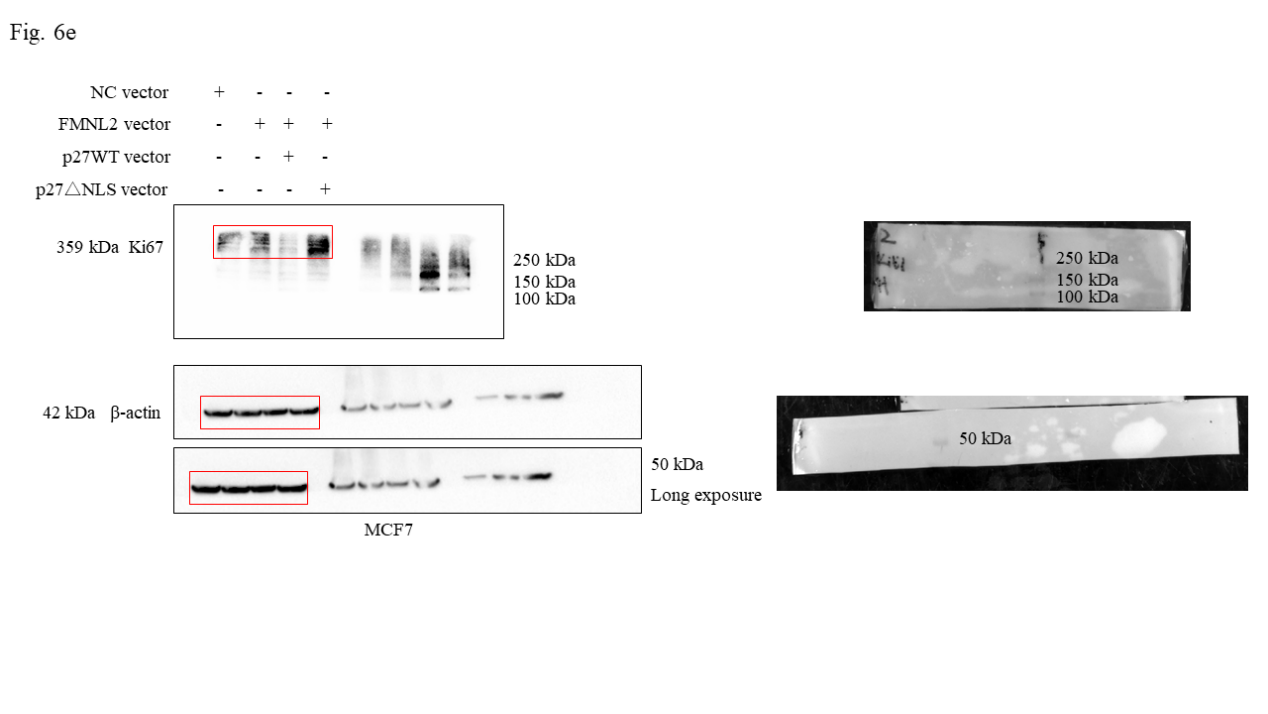


**Fig.6** FMNL2 promotes breast cancer cell proliferation partially by a p27-related mechanism. After transfection for 48 h, cultured cells were processed for indicated assays. **a** and **b** The expression of FMNL2, p27 and Ki67 was detected by western blotting. **c** MTT assay was performed at 48 h post-transfection. *: *P*<0.05 *vs* NC siRNA group. ^#^: *P*<0.05 *vs* sip27 group. **d** and **e** The expression of FMNL2, p27, Lamin A/C and Ki67 was detected by western blotting. **f** MTT assay was performed at 48 h post-transfection. **g** Representative percentage of cells in the G0/G1, G2/M and S phases was detected by ﬂow cytometric analysis. *: *P*<0.05 *vs* p27WT vector group.
